# Supramolecular arrangement of the full-length Zika virus NS5

**DOI:** 10.1371/journal.ppat.1007656

**Published:** 2019-04-05

**Authors:** Diego S. Ferrero, Victor M. Ruiz-Arroyo, Nicolas Soler, Isabel Usón, Alba Guarné, Núria Verdaguer

**Affiliations:** 1 Structural Biology Unit, Institut de Biología Molecular de Barcelona CSIC, Barcelona, Spain; 2 ICREA Institució Catalana de Recerca i Estudis Avançats, Barcelona, Spain; 3 Department of Biochemistry, McGill University, Montreal, Quebec, Canada; Institut Pasteur, FRANCE

## Abstract

Zika virus (ZIKV), a member of the *Flavivirida*e family, has emerged as a major public health threat, since ZIKV infection has been connected to microcephaly and other neurological disorders. Flavivirus genome replication is driven by NS5, an RNA-dependent RNA polymerase (RdRP) that also contains a N-terminal methyltransferase domain essential for viral mRNA capping. Given its crucial roles, ZIKV NS5 has become an attractive antiviral target. Here, we have used integrated structural biology approaches to characterize the supramolecular arrangement of the full-length ZIKV NS5, highlighting the assembly and interfaces between NS5 monomers within a dimeric structure, as well as the dimer-dimer interactions to form higher order fibril-like structures. The relative orientation of each monomer within the dimer provides a model to explain the coordination between MTase and RdRP domains across neighboring NS5 molecules and mutational studies underscore the crucial role of the MTase residues Y25, K28 and K29 in NS5 dimerization. The basic residue K28 also participates in GTP binding and competition experiments indicate that NS5 dimerization is disrupted at high GTP concentrations. This competition represents a first glimpse at a molecular level explaining how dimerization might regulate the capping process.

## Author summary

The lack of vaccine or antiviral drugs to combat Zika virus (ZIKV) infection has encouraged scientists to characterize in depth potential drug targets. One attractive candidate is NS5, responsible for the catalysis of the 5’-RNA capping, methylation and RNA synthesis, during flavivirus genome replication. To fulfill these activities, the methyltransferase and RNA-dependent RNA polymerase domains of NS5 need to cooperate with each other. The structural and biophysical data presented in this work demonstrate that the ZIKV NS5 protein has the ability to form dimers, as well as higher order oligomers that may participate in the fine-tuning regulation of the multiple enzyme functions in the replication complex. In addition, we have found that NS5 dimerization is disrupted at high GTP concentrations, explaining how dimerization might regulate the capping process.

## Introduction

Zika virus (ZIKV) is a mosquito-borne flavivirus, closely related to other important human pathogens such as Dengue (DENV), West Nile (WNV), Japanese encephalitis (JEV) and yellow fever viruses (YFV) [1]. First isolated in 1947 from a sentinel rhesus macaque in the Zika Forest region of Uganda [2], ZIKV remained neglected for many years until outbreaks occurred on Yap island in 2007 and within the French Polynesian islands in 2013–2014, before spreading across the Pacific Ocean and probably invading South America [3–6]. After the large Brazilian outbreak started in late 2014, active transmission was reported in approximately 60 countries and territories globally [7]. Contrasting the Yap island outbreak, which was characterized by cases with relatively mild dengue-like symptoms the outbreaks in French Polynesia and Brazil were associated with an unusual proportion of serious neurological disorders such as microcephaly in newborn infants [8] and Guillain-Barré syndrome in adults [9–10]. The public-health emergency raised by ZIKV [11] stimulated efforts to build up human *in vitro* and animal *in vivo* models to understand how ZIKV causes developmental abnormalities [12–13], and to develop a vaccine. The contribution of structural biology to the elucidation of the molecular mechanisms of ZIKV infection has been spectacular, with the structural descriptions of most of the soluble viral proteins, domains and assemblies becoming available in less than one year, reviewed in [14]. However, the understanding of how the different proteins and/or protein domains interact with each other to form functional complexes is still limited.

As a member of the Flaviviridae family, ZIKV has a single-stranded positive sense RNA genome (~11 Kb), harboring a conserved type 1 RNA cap (m7GpppA2’OmG) at the 5’-end. Upon infection, the genome is translated to a single polyprotein that is post-translationally cleaved into three structural proteins (C, PrM/M and E) and seven nonstructural (NS) proteins (NS1, NS2A, NS2B, NS3, NS4A, NS4B and NS5). The NS proteins assemble with an array of host factors into membrane-bound replication complexes where viral RNA synthesis takes place [15]. Among them, the multi-task protein NS5 plays crucial roles. It comprises an N-terminal S-adenosyl-L-methionine (SAM)-dependent methyltransferase (MTase) domain and an RNA-dependent RNA polymerase (RdRP) domain at the C-terminus. The N-terminal MTase adopts the classical Rossmann fold, composed of six helices surrounding a central 7-stranded β-sheet and possesses a cap binding pocket where the cap guanosine is held during 2’-O-methylation by stacking interactions with a phenylalanine residue conserved in all flaviviruses [16]. The C-terminal RdRP domain adopts the canonical right-hand polymerase fold, consisting of fingers, palm, and thumb subdomains, with the catalytic GDD motif located in the palm and a long priming loop protruding into the active site cavity. Although MTases are common for viruses bearing a 5’ cap structure and RdRP are found in all RNA viruses, the flavivirus NS5 represents a unique natural fusion of these two important enzymes. The longstanding question, however, has been whether the two domains cooperate to regulate the viral replication activity.

The NS5 structures of the closely related flaviviruses JEV and DENV have been previously determined, showing important differences in the MTase and RdRP relative orientations and the inter-domain interfaces between the two viruses [17–19]. In addition, while this manuscript was in preparation the crystal structures of the full length ZIKV NS5, strain MR776, have been reported by three different groups [20–22] (PDBs: 5TFR; 5TMH; 5U0B), revealing striking similarities to the NS5 protein of JEV [17] (PDB: 4K6M). Here we report the X-ray structures and quaternary organization of the Suriname ZIKV human isolate Z1106033 full-length NS5. The protein crystallized in two different space groups, hexagonal P6_5_ and orthorhombic P2_1_2_1_2_1_ both containing six independent NS5 molecules in the crystal asymmetric unit organized in three quasi-equivalent dimers. Dimer-dimer interactions were also conserved to form a higher-order helicoidal structure. A monomer-dimer equilibrium was confirmed in solution by size exclusion chromatography associated to multiangle light scattering (SEC-MALS), analytical ultracentrifugation (AU), small angle X-ray scattering (SAXS) and cross-linking. A fibril-like morphology of ZIKV NS5 was also observed by negative staining transmission electron microscopy (TEM) and atomic force microscopy (AFM) imaging. These structures shed new light into the key interactions that would contribute to the regulation of the different enzymatic activities, providing the foundation to start developing a new generation of antivirals targeting functional intermolecular interfaces.

## Results

### Overall structure of the Suriname ZIKV full length NS5

We produced the recombinant NS5 protein (residues 1–904) from Suriname ZIKV human isolate Z1106033 in *Escherichia coli*. The recombinant his-tagged NS5 protein was purified to homogeneity and then subjected to multiple crystallization trials. Two different crystal forms, orthorhombic P2_1_2_1_2_1_ and hexagonal P6_5_, were obtained, containing six independent NS5 molecules in each asymmetric unit. The best diffracting crystals were obtained after applying a soft dehydration protocol by soaking them in a harvesting buffer that included the reservoir solution, with a 5% increase of the phosphate salts. Anisotropic diffraction was observed, particularly in the P2_1_2_1_2_1_ crystals and correction was applied during data processing (see Methods and S1 Table).

**Fig 1:**
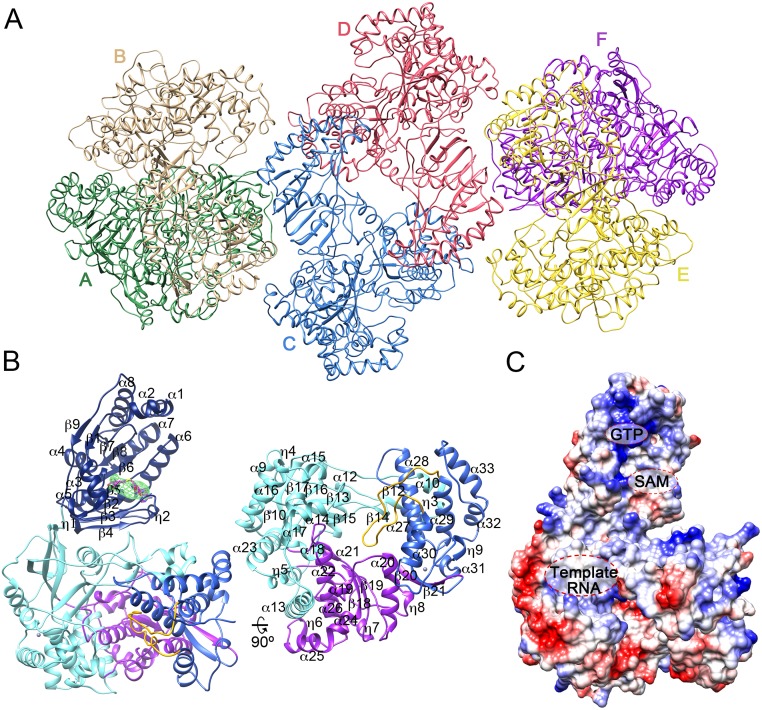
Overall views of ZIKV NS5 structure and quaternary arrangement. **(A)** Cartoon representation of the crystallographic asymmetric unit corresponding to the P2_1_2_1_2_1_ crystal, containing six monomers, labeled A to E (A, green; B, light brown; C, blue; D, red; E, yellow; F, purple) that assemble into three homodimers. **(B)** Cartoon representation of one NS5 monomer shown in an orientation, looking from the top of RdRP (left panel). Secondary structural elements are explicitly labeled and numbered. The MTase is shown in dark blue with the bound SAH molecule shown as sticks in atom type code with carbon atoms in light red. The difference Fo-Fc electron density map (3σ) is shown as a green mesh. The RdRP fingers, palm and thumb sub domains are depicted in cyan, purple and blue, respectively, with the priming loop protruding from the thumb highlighted in yellow. The right panel shows a view of the RdRP domain, looking into the RdRP front channel. **(C)** Surface representation of the protein with the electrostatic potential colored in blue and red for positive and negative charges, respectively. The GTP and SAM/SAH binding sites in the MTase domain, and the RNA template channel in the RdRP are explicitly labeled.

The structures were solved by molecular replacement, using two independent search models derived from the ZIKV MTase (PDB: 5KQR, [23]) and JEV RdRP (PDB: 4K6M, [17]) domains. In the two crystal forms, the six independent NS5 molecules were organized in three quasi-equivalent dimers (Fig 1A). Despite the limited resolution (4 Å and 4.8 Å resolution for the P2_1_2_1_2_1_ and P6_5_ crystals, respectively), the resulting electron density maps not only showed a perfect fitting of the two molecular replacement models but also allowed the tracing of bulky side chains (S1 Fig), as well as the ten amino acids linker (residues 263–272), joining the MTase to the backside of the RdRP domain in four out of the six molecules of the asymmetric unit. The high solvent content of these crystals (80% and 77% for the P2_1_2_1_2_1_ and P6_5_ crystals, respectively) significantly helped to improve the maps using density modification. Extra difference densities were also seen in the SAM/SAH binding pockets to accommodate the ligands in identical positions to those previously reported [17,18,23], although none of these compounds were added to the crystallization solutions (Fig 1B and S1 Fig).

The ZIKV NS5 polypeptide adopts an extended fold in which the MTase domain (residues 1–262) is placed above the fingers sub-domain, behind the RdRP (residues 273–888), opposite to the double stranded RNA exit channel and close to the template binding channel (Fig 1B and 1C). In this conformation, the SAM-binding and cap-binding sites of the MTase are fully accessible in an orientation that appears to complete the template binding channel of the RdRP. This conformation is conserved in the twelve independent molecules determined in this work, as well as in the two independent molecules contained in the asymmetric units of ZIKV, strain MR776 (PDBs: 5TFR, 5TMH and 5U0B) [20–22], with root mean square deviation (rmsd) values ranging from 0.1 Å to 0.6 Å and hinge rotations of 3° to 5° for the superimposition of all Cα atoms (S2 Fig). This global conformation is also closely related to that previously reported for JEV NS5 [17].

Sequence alignments showed that the Suriname ZIKV human isolate Z1106033 NS5 sequence differs by 31 amino acids from its counterpart in the MR776 strain (S3 Fig). Most of these residues are exposed at the protein surface and 9 of them are directly involved in crystal packing interactions in the small P2_1_2_1_2 unit cell, corresponding to the previous reported structures that contain two independent NS5 molecules in the asymmetric unit [20–22]. These differences might explain the different packing preferences of the two NS5 proteins.

### Quaternary arrangement of ZIKV NS5 in crystals

**Fig 2:**
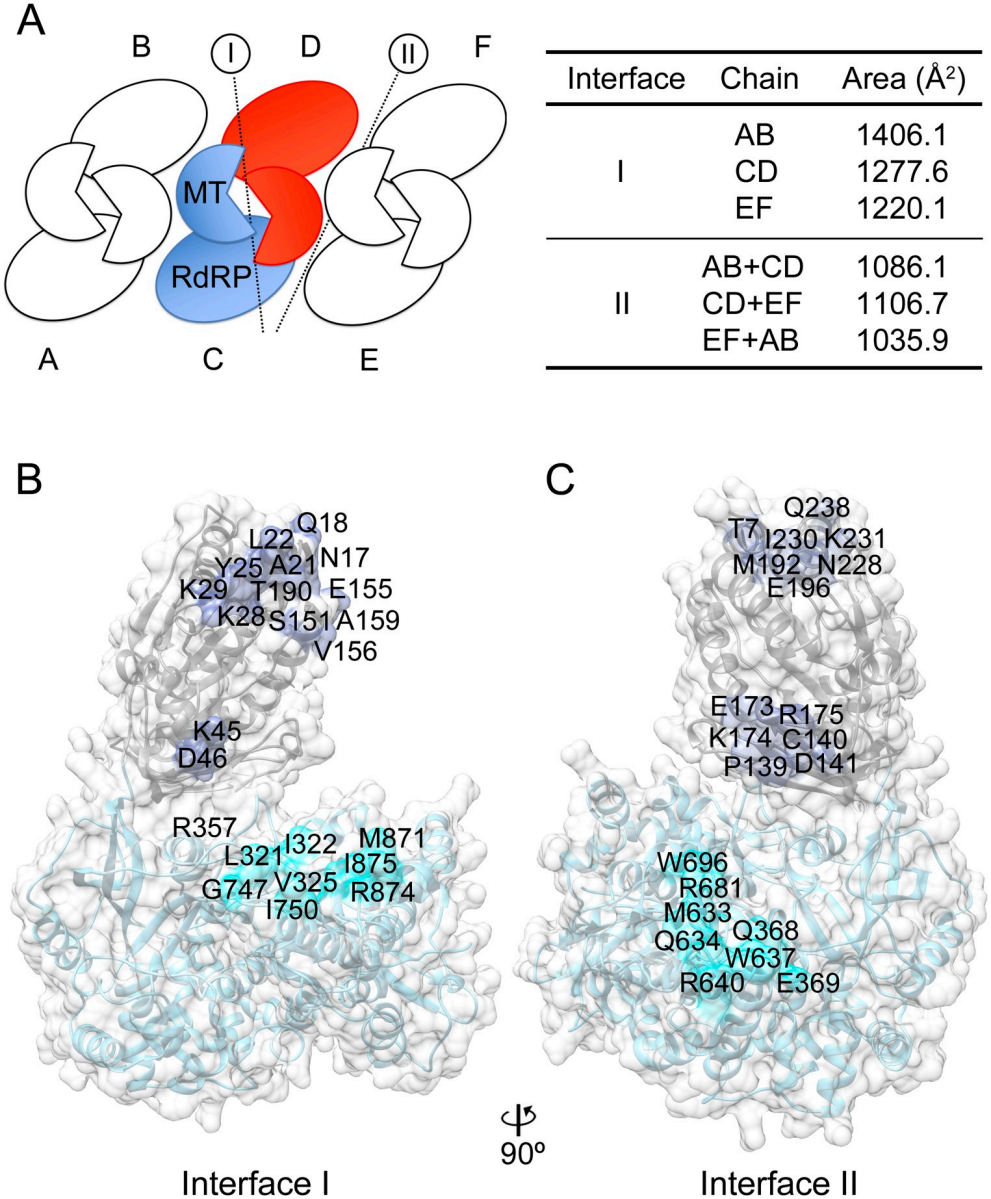
NS5 dimer interactions. **(A)** Schematic representation the arrangement of the three ZIKV NS5 dimers present in the asymmetric unit of the P2_1_2_1_2_1_ crystals (left panel). The reference monomers are shown in red and blue. The buried surface areas are summarized in the table (right panel). Interfaces I and II indicate the monomer-monomer and dimer-dimer interactions, respectively. **(B)** Surface representation of NS5. Residues involved in interface I monomer-monomer contacts are explicitly labeled. The color code indicates the position of the interacting residues in the surface of the RdRP (cyan) or the MTase (light blue) domains. (**C**) Surface representations of the ZIKV, highlighting residues involved in dimer-dimer contacts (interface II). As in **B**, color codes indicate the interacting residues.

**Fig 3:**
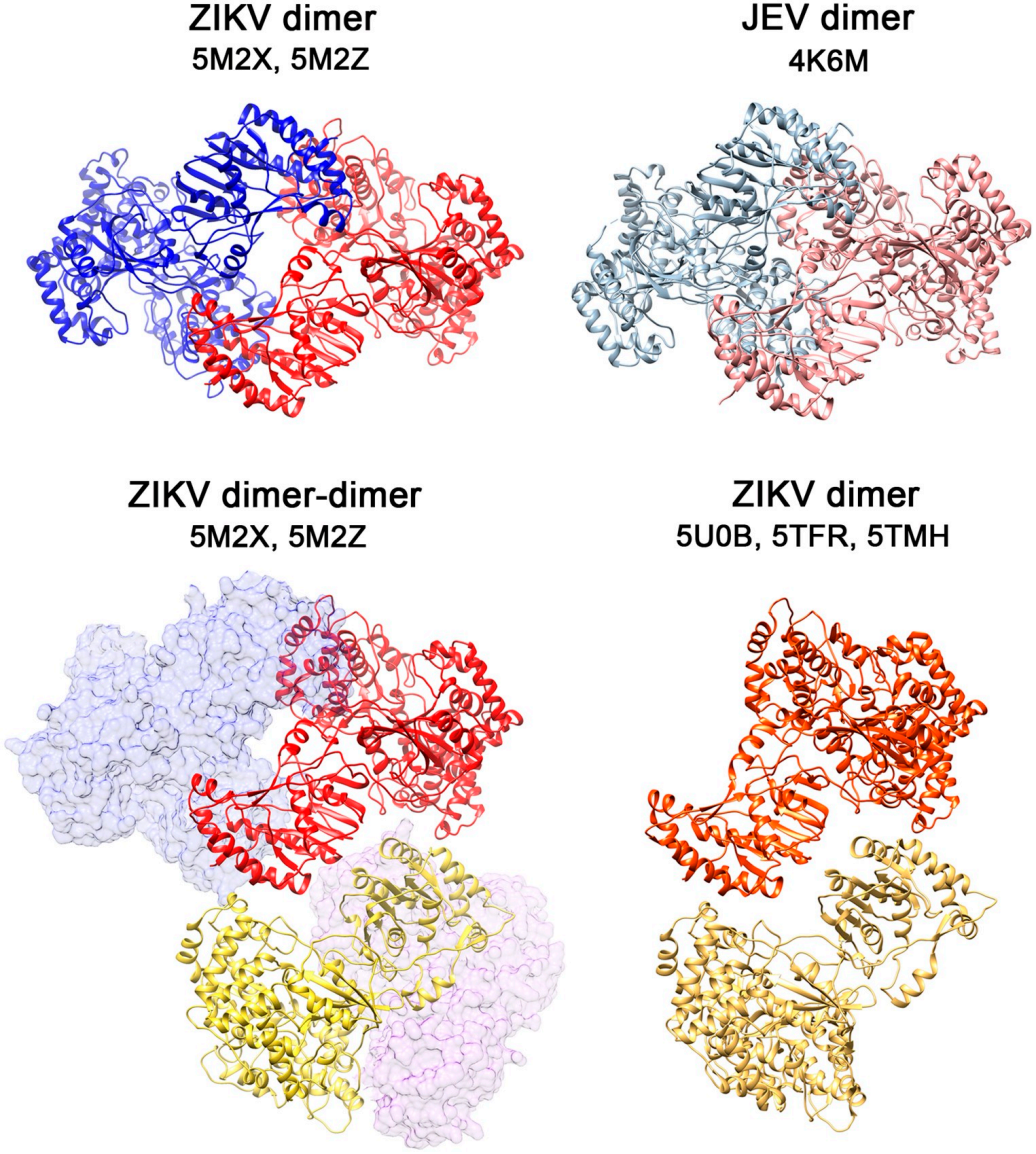
Arrangement of the NS5 dimers. Cartoon representation of the ZIKV NS5 dimers observed in the P2_1_2_1_2_1_ (PDB: 5M2X) and P6_5_ (PDB: 5M2Z) crystals (this work), with one monomer shown in blue and the second in red (top left panel). For comparison, the arrangement of the JEV NS5 dimer (PDB: 4K6M) [17] is shown in the top right panel. The bottom left panel shows the conserved dimer-dimer arrangement of ZIKV NS5 seen in the crystal asymmetric units of both P2_1_2_1_2_1_ and P6_5_ crystal forms. The two interacting monomers are shown as cartoons in red and yellow and the partner molecule to form each dimer are depicted as semitransparent surfaces. The arrangement of the two monomers of ZIKV NS5, strain MR776 (PDBs: 5TFR; 5TMH; 5U0B) [20–22] is shown on the bottom right panel (interacting monomers are in orange and wheat).

The crystallographic asymmetric unit of both crystals consists of three dimers (named AB, CD and EF in P2_1_2_1_2_1_ and A’B’, C’D’ and E’F’ in P6_5_ crystals; Fig 1A), in which the monomers are related by pseudo-twofold molecular axes (Fig 2A). Each monomer contributes >1200 Å^2^ (30% of the total surface) to the dimer interface (Fig 2A and 2B). The interface of interaction (interface I) involves two types of intermolecular contacts, MTase-MTase and MTase-RdRP. MTase-MTase interactions involve the periphery of the MTase in one NS5 molecule: helices α1(N17, Q18), α2 (A21, L22, Y25, K28) and the α2-β1 loop (K29) which contact residues K45 and D46 (α3) of the second NS5 subunit (Fig 2B). MTase-RdRP contacts include the MTase α6 residues, E155, V156, A159 of the first monomer which contact two RdRP subdomains of the neighbor NS5 involving residues: a) L321, I322, V325 (β11) and G324,V325 (α10) within fingers and b) G747, I750 (β22-α28 loop) and M871, R874 and I875 (α33) at the back of the thumb (Fig 2B). In this arrangement the MTase active site cavities of the two interacting molecules are directly connected and the two RdRP exit channels remain accessible to the solvent. The six dimers can be superimposed with rmsd values ranging from 0.8 Å to 1.0 Å for the superimpositions of all 1,776 equivalent Cα atoms. Comparable head-to-tail dimers were also observed in the structure of JEV NS5 (PDB: 4K6M) [17] but showing a slightly different rotation between monomers (Fig 3).

Dimer-dimer interactions (Interface II; Fig 2A and 2C) are also conserved in both ZIKV NS5 crystal forms and involve contacts between molecules B-C and D-E (P2_1_2_1_2_1_ crystals) and B’-C’ and D’-A” (P6_5_ crystals, with A” corresponding to a symmetry related A’ molecule). Three distinct NS5 regions are implicated in interface II contacts, burying >1000 Å^2^ of surface area between monomers in each pair (Fig 2A and 2C, S4 and S5 Figs). Similar contacts were observed between the two molecules contained in the asymmetric unit of ZIKV MR776, NS5 crystals (PDBs: 5TFR; 5TMH; 5U0B) [20–22] (Fig 3).

### SEC-MALS and AUC experiments show that NS5 exists as a monomer-dimer equilibrium in solution

**Fig 4:**
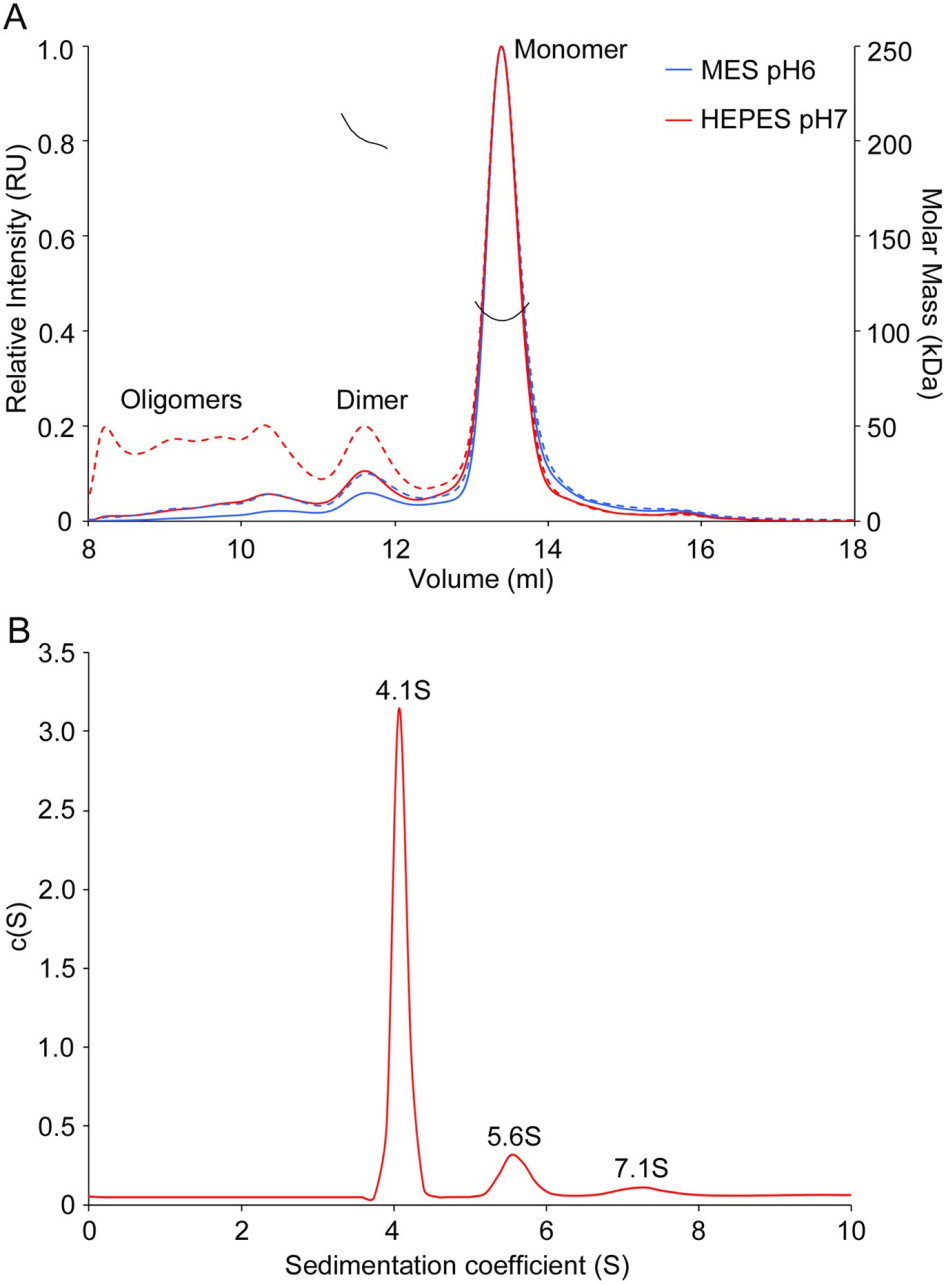
Solution studies of NS5 by SEC-MALS and AUC. **(A)** SEC-MALS profiles of ZIKV NS5 in 50 mM MES pH 6.0, 500 mM NaCl, 10% glycerol, 5 mM DTT buffer (blue solid line) and 50 mM HEPES pH 7.0, 500 mM NaCl, 10% glycerol, 5 mM DTT (red solid line). Dashed lines represent light scattering for each condition and black traces show the measured molecular masses (right axis) of protein in the corresponding peak. (B) Sedimentation coefficient distribution profile of NS5, indicating the values for each peak that would correspond to monomer (4.1S), dimer (5.6S) and oligomer (7.1S) species. Sedimentation coefficients (S) are expressed in Svedberg units (1S = 10^-13^m s^-1^) and c(S) corresponding to normalized continuous size distribution.

Size exclusion chromatography (SEC) analysis of the purified NS5 protein in the storage buffer (50 mM MES pH 6.0, 500 mM NaCl, 10% glycerol, 5 mM DTT) revealed the presence of a predominant peak (95 ± 2% of the mass sample) corresponding to a 104 ± 2 kDa molecule determined by a coupled MALS. This value perfectly matches the molecular mass of the NS5 monomer. A second peak, representing 4 ± 2% of the sample mass, could also be quantified by light scattering (178 ± 2 kDa). This second peak would correspond to the NS5 dimeric form (Fig 4A). Similar results were obtained with an alternative NS5 stabilization buffer (50 mM HEPES pH 7.0, 500 mM NaCl, 10% glycerol, 5 mM DTT) (Fig 4A), the predominant peak (89.5 ± 0.1%) with a MW of 105.8 ± 0.3 kDa eluted after a less abundant species. One of them, with 202.3 ± 0.4 kDa and 10.4 ± 0.1% of abundance was clearly associated to a dimeric form of NS5, while the molecular weights of other aggregates visible in HEPES buffer were not measurable.

Further studies of the NS5 quaternary state were performed by sedimentation velocity in AUC at fixed protein concentration (0.5 mg/ml). Three species were identified: i) a predominant single species (82.9%) with a sedimentation coefficient of 4.1S and a frictional ratio (f/f0) of 1.49, compatible with an ellipse with a radius of 8.96 nm that would correspond to the NS5 monomer; ii) a second species (12% abundance), compatible with the mass of a dimer (208.35 kDa), had a sedimentation coefficient of 5.7S and a f/f0 value of 1.69, showing an ellipsoidal shape with a maximum radius of 12.92 nm; iii) the remaining species were detected as a broad pick representing the 5.1% of the sample and would correspond to an oligomer with a mean sedimentation coefficient of 7.12S, but could not be analysed further. Consistently with the SEC-MALS data, the sedimentation velocity results showed a similar distribution of protein species within the sample (Fig 4B and S6 Fig).

### SAXS data confirm NS5 monomer-dimer equilibrium in solution

**Fig 5:**
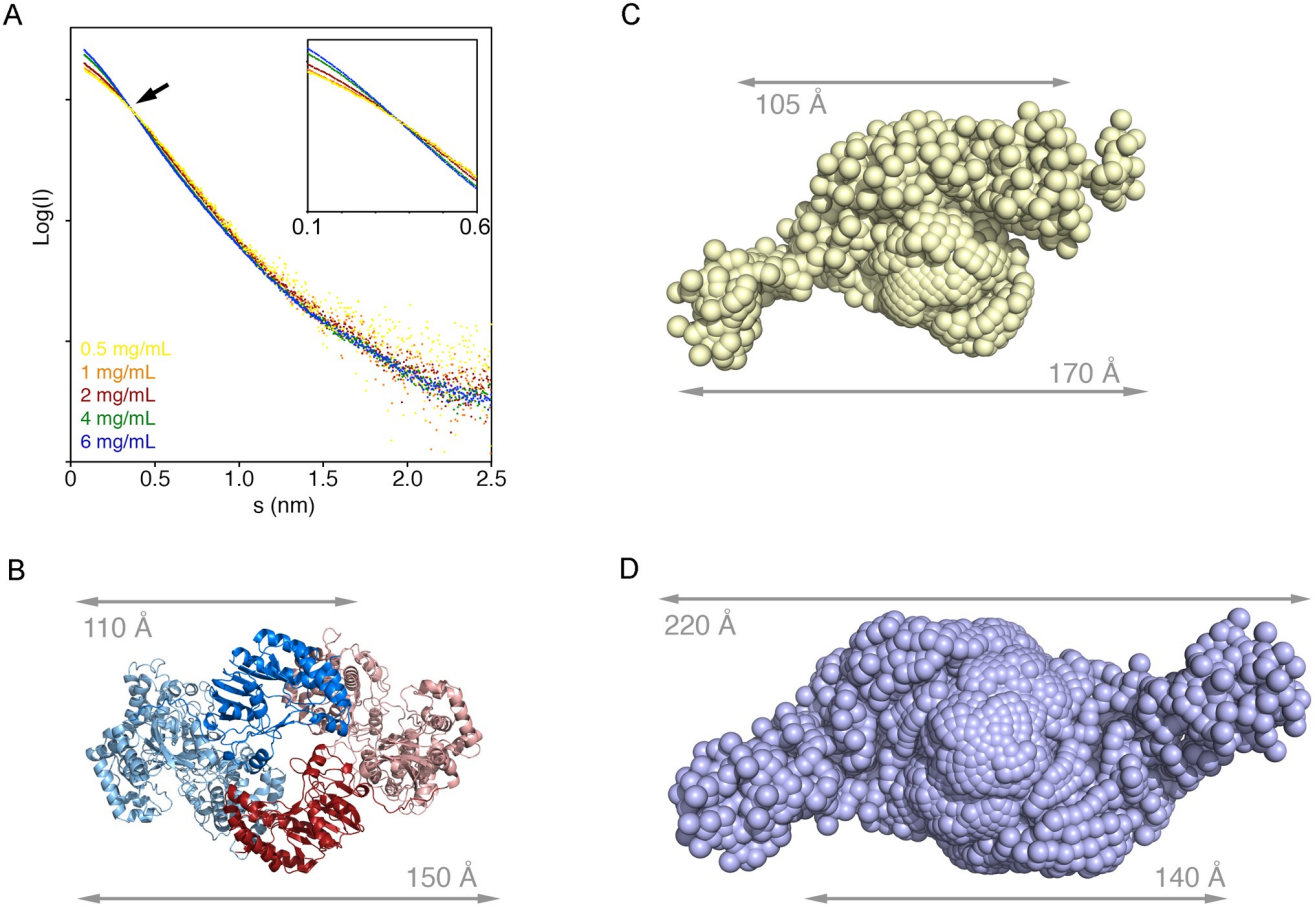
Solution studies of NS5 by SAXS. **(A)** Scattering curves at increasing concentrations of NS5. The curves collected at low (0.5 mg/ml) and high (6 mg/ml) NS5 concentrations cross each other indicating the presence of different scattering species. The inset shows a detail of the small angle region of the curves. **(B)** Ribbon diagram of the NS5 dimer with the protomers colored in blue and red, respectively, highlighting the MTase domains with solid colors. The dimensions of the monomer and the dimer are indicated. **(C-D)** *Ab initio* bead models generated with Gasbor corresponding to the scattering curves collected at 0.5 mg/ml **(C)** and 6 mg/ml **(D)**.

To visualize the NS5 dimer being formed in solution, we used SAXS to calculate the experimental maximum particle diameter (D_max_) and molecular weight (MW) from samples at increasing protein concentrations (0.5–6 mg/ml). The experimental molecular weight of NS5 at the lowest concentration was consistent with the calculated molecular weight of a monomer. However this value increased in a concentration dependent manner and at 6 mg/ml, it was consistent with the presence of a dimer in solution (Fig 5A). The Guinier plots for all concentrations were of excellent quality and devoid of interparticle interactions, indicating that the increase in molecular weight was not due to protein aggregation. Similarly, the Kratky plots indicated that all samples were properly folded (S7 Fig). We noted that the scattering curves at increasing protein concentrations intersected (Fig 5A), which is indicative of a titration between two species. Given the excellent quality of all samples and the molecular weights calculated from each curve (S2 Table), we proceeded to calculate *ab initio* models of the lowest (corresponding to the NS5 monomer) and highest (corresponding to the NS5 dimer) concentration samples. Both models yielded excellent fits to the scattering curves (chi^2^ of 0.98 and 4.4, respectively), but were larger than the crystallographic monomer and dimer (Fig 5C and 5D). SAXS measurements at the lowest and highest protein concentrations resulted in bell-shaped pair-distance distribution functions with a trailing tail (S7 Fig), strikingly similar to those previously obtained for the monomer and dimeric forms of NS5 by Saw and co-workers [24].

At 0.5 mg/ml, the bell-shaped distribution extends from 0-120Å with the tail extending to 170 Å (S7 Fig). This distribution indicates that only few interatomic distances are that long, in turn indicating that the monomer may have a flexible region that extends beyond the folded domain. The model derived from the crystal structure includes residues 6–888 of NS5 and has 26 amino acids disordered at the C-terminus (five from the protein C-terminus plus the His-tag and the spacer). The disordered C-terminal tail is readily modeled in an extended conformation on the EOM models, reconciling the differences between the dimensions obtained from the crystal structure and the scattering curves. Similarly, the pair-distance distribution function for the sample at 6 mg/ml (at which concentration NS5 exists as a dimer), the bell-shaped distribution extends from 0-170Å with the tail extending to ~220 Å (S7 Fig). The elongated dimer observed in solution is probably caused by a combination of the volume occupied by the disordered residues in the crystal structure and small distortions of the crystallographic dimer when devoid the crystal packing constrains.

The 10-amino acid linker connecting the MTase and RdRP domains of Flavivirus NS5 proteins, would assist the conformational flexibility of the molecule as previously seen in Dengue virus NS5 [25–26]. To explore whether this linker also enables the flexibility of Zika NS5, we analyzed the scattering data for the lowest concentration of NS5 (0.5 mg/ml) using EOM [27–28]. This analysis confirmed that the linker is mobile (chi^2^ of 2.4), but not completely flexible because the EOM sub-ensemble that best described the low concentration data was populated by only two conformations. Both conformations were more extended than the crystallographic monomer indicating that, similarly to dengue NS5, Zika NS5 also adopts an extended conformation in solution [26]. The extended conformation of the model was retained at all concentrations analyzed (Fig 5C and 5D), confirming that the dimer may retain the conformational flexibility of the monomer.

### The MTase-MTase contacts drive NS5 dimerization

The full length NS5 as well as the individual MTase and RdRP domains were subjected to cross-linking with the label-free cross-linker Bis (sulphosuccinimidyl) suberate (BS3, ThermoFisher Scientific). The analysis of the cross-linked complexes by SDS-PAGE showed that full length NS5 and the MTase domain are able to form dimers, as well as higher-order oligomers in solution, whereas the RdRP domain is monomeric (S8 Fig).

**Fig 6:**
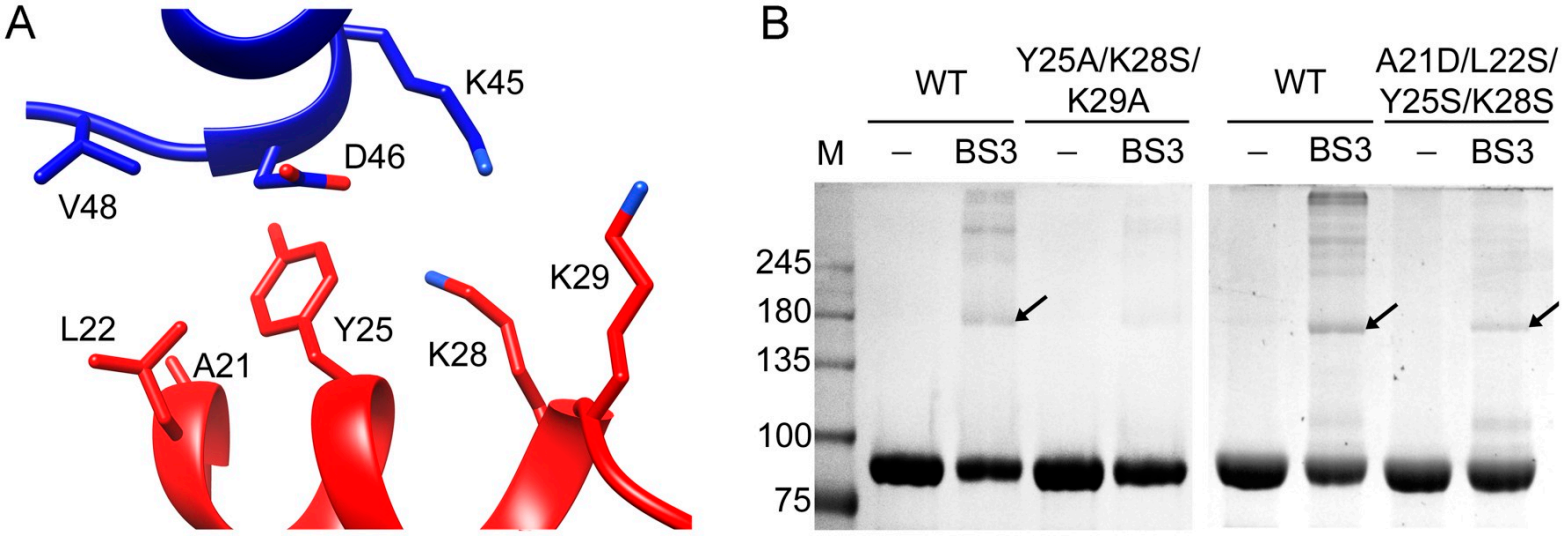
MTase-MTase contacts are the main players in NS5 dimer formation. **(A)** Detail of the main MTase-MTase interactions, involving the α2 residues (red) of one protomer with the α3 residues (blue) of the second protomer. **(B)** SDS-PAGE of the ZIKV NS5 wild type, Y25A/K28S/K29A and A21D/L22S/Y25A/K28S mutant proteins. Proteins (5 μM) are crosslinked with 10 μM BS3 (45 min incubation) in buffer 20 mM HEPES pH 7.0, 150 mM NaCl. The formation of dimers (black arrow) is almost abolished in the NS5 Y25A/K28S/K29A mutant.

The crystal structures showed that the major MTase-MTase contact interface involved the α2 helix residues A21, L22, Y25 and K28, and K29 (α2-β1 loop), which are involved in polar and hydrophobic intermolecular contacts with the α3-helix residues from K45 to V48 (Figs 2B and 6A). To confirm the role of α2 residues as the main NS5 dimerization surface, we generated two different mutants with changes at the contacting area, (A21D/L22S/Y25S/K28A and Y25A/K28S/K29A) and tested their effect on dimer formation by BS3 crosslinking and SDS-PAGE analysis. As shown in Fig 6B, the motif Y25/K28/K29 plays an essential role, because substitutions in these amino acids result in an important decrease in dimer formation.

### ZIKV NS5 fibers seen by both negative staining EM and AFM

**Fig 7:**
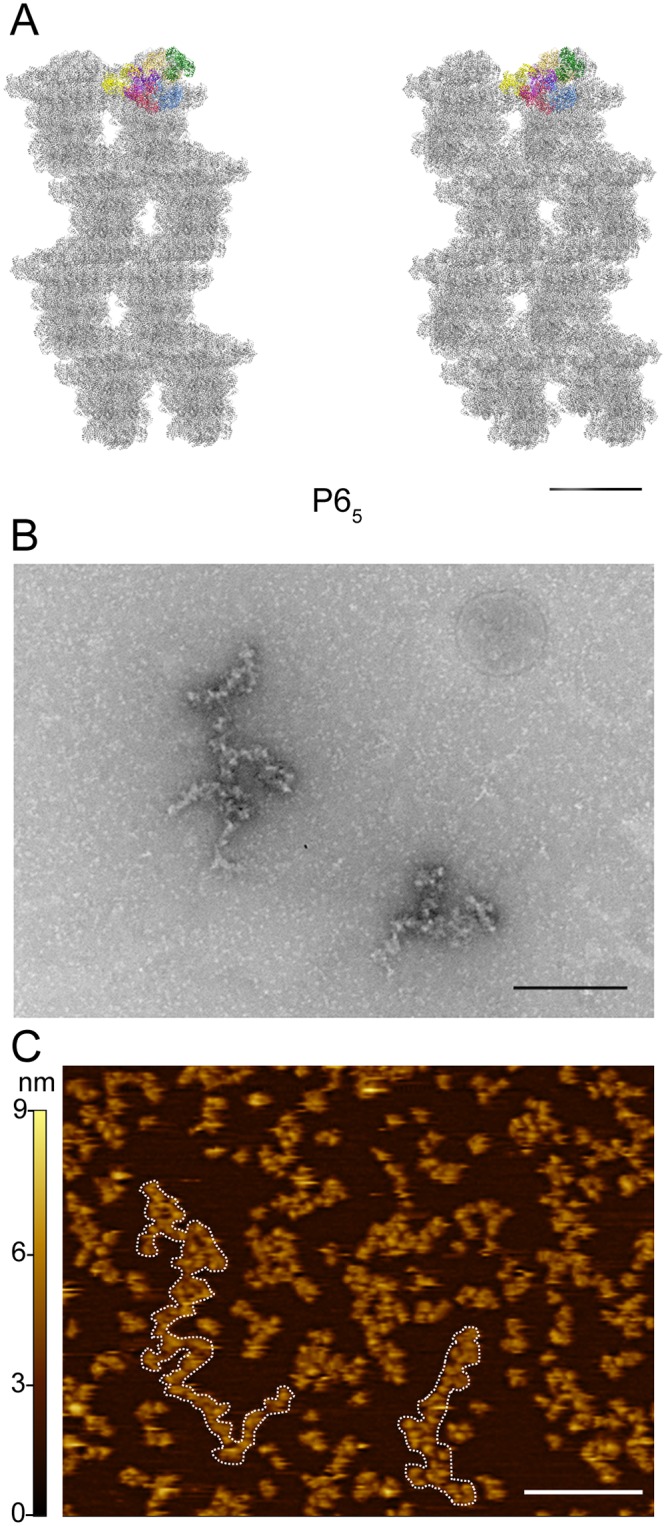
ZIKV NS5 fibers observed by negative staining TEM and AFM. **(A)** Stereo view of the quaternary arrangement of ZIKV NS5 along the unit cell c axis in the P6_5_ crystal, highlighting the content of the asymmetric unit (Chains: A, green; B, light brown; C, blue; D, red; E, yellow; F, purple). The scale bar corresponds to 200 Å. **(B)** Selected area from an electron micrograph of a negative stained sample (2% uranyl acetate) showing particles of NS5 disposed in a fibril-like arrangement in buffer 50 mM MES pH 6.0, 50 mM NaCl, 5 mM DTT. The scale bar corresponds to 250 nm. **(C)** AFM topography in the protein buffer (50 mM MES pH 6.0, 500 mM NaCl, 10% glycerol, 5 mM DTT). White scale bar corresponds to 100 nm. The left lateral scale in nm corresponds to the z-axis describing the height of the scanned surface (0.5x0.5μm) by an artificial color scale. White doted lines highlight a couple of string-like oligomers of NS5.

The conserved dimer-based helicoidal structures observed in both crystal forms (Fig 7A) involving significant intermolecular contact areas (> 1000 A^2^), suggest that these assemblies would be stable and might exist in solution and possibly in cells as part of the viral replication complexes. In fact, negative staining EM imaging confirms the presence of NS5 derived fibril-like oligomers, in different experimental conditions, including low and high ionic strength buffers (containing 50 mM and 250 mM NaCl) and protein concentration (Fig 7B and S9A–S9C Fig). The tendency of the wild type ZIKV NS5 to form elongated fibril-like arrangements is abolished in the NS5-Y25A/K28S/K29A variant, even at high protein concentration (S9D Fig).

Furthermore, tapping mode AFM imaging of NS5 in the protein buffer solution (50 mM MES pH 6.0, 500 mM NaCl, 10% glycerol, 5 mM DTT), adsorbed onto mica reveals arrangements of NS5 like strings made up of globular particles connected side by side, similar to that seen by negative staining EM (Fig 7C and S10A–S10C Fig). AFM topographies showed the diversity of these fibril-like structures with 3–3.5 nm height, a variable length ranging from 20 to 300 nm and 15–25 nm width (S10E–S10G Fig). Although the width value is influenced by the tip size, driving to overestimation of the real size, the estimated values are not far from those measured in the X-ray structure of the type I dimers (S10E–S10G Fig). As expected, AFM imaging of the NS5-Y25A/K28S/K29A variant did not show the presence of fibril-like structures (S10B–S10D Fig).

### *In vitro* polymerase activity of recombinant ZIKV NS5 proteins

**Fig 8:**
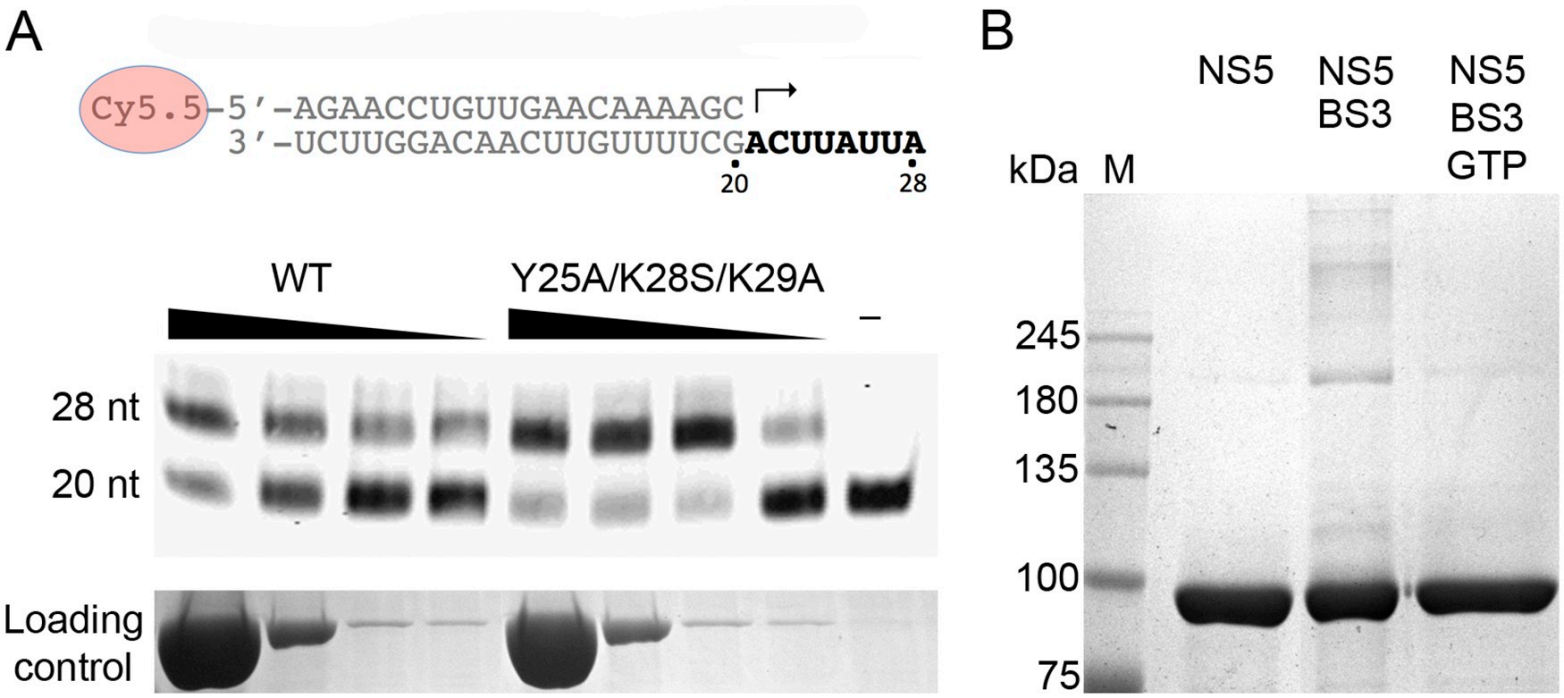
Effects of dimerization in NS5 functions. **(A)** Polymerase activity evaluated in a nonradioactive primer extension assay. The top panel shows the template/primer duplex used in the assay. The arrow indicates the location and direction of primer extension. The bottom panel shows the effect of the wild type NS5 or NS5-Y25A/K28S/K29A dimerization mutant concentrations (900, 180, 18, 9 nM) on polymerization activity. This assay used a constant template/primer concentration of 10 nM. **(B)** SDS-PAGE of the ZIKV NS5 wild type protein (5 μM) crosslinked with 10 μM BS3 (45 min incubation), in buffer 20 mM HEPES pH 7.0, 150 mM NaCl, in the absence or presence of 50 mM GTP.

The *in vitro* RNA synthesis activities of the purified wild type NS5 and NS5-Y25A/K28S/K29A variant were analyzed in a primer extension assay, using a fluorescently labeled RNA primer (20-mer) annealed to an unlabeled RNA template (28-mer), following a previously described protocol [29]. The presence of the full-length 28-mer primer extension RNA product in denaturing polyacrylamide gel electrophoresis indicated that the primer was fully extended by both proteins in a reaction dependent of Mn^2+^ as catalytic ion (Fig 8A). To compare the enzymatic activity of wildtype and NS5-Y25A/K28S/K29A variant, an enzyme dilution experiment was carried out using different protein concentrations. As shown in Fig 8A, the NS5-Y25A/K28S/K29A variant is about three times more active than the wild type NS5 at higher protein concentrations where quaternary structure is promoted, suggesting that NS5 dimerization downregulates the RdRP elongation activity.

### GTP perturbs ZIKV NS5 dimerization

The crystal structures showed that the MTase α2 residues A21, L22, Y25 and K28, and K29 (α2-β1 loop) constitute a major MTase-MTase contact interface contributing to dimer stabilization and our mutagenic studies confirmed the key role of amino acids Y25/K28 /K29 in NS5 dimerization. The conserved basic residue K28 has also been shown to participate in the GTP-binding pocket, as seen in the structures of different flavivirus MTases that have been crystallized in presence of this nucleotide [16, 30–31]. The possible role of this residue in GMP binding during guanylyltransferase (GTase) activity of flavivirus NS5 was described earlier [30, 32] and evidence of a covalent linkage between the K28 residue from the Wesselsbron virus NS5 and GMP were previously provided [30]. As K28 appeared to be involved in both GTP binding and NS5 dimerization, we decided to study the relationship between the two activities by performing BS3 cross-linking experiments of NS5 in presence/absence of GTP. Data reveal that NS5 dimer/oligomer formation was hindered in presence of this nucleotide (Fig 8B). To further confirm that this was a guanine-specific effect, cross-linking experiments were also performed in presence of ATP, as negative control and with GMP. These experiments showed that though dimers remain in presence of ATP, the GMP molecule had almost the same effect as GTP (S11 Fig). These results suggest that NS5 dimerization might negatively regulate the GTase activity of NS5, a crucial step in the capping process.

## Discussion

Genome replication in flaviviruses, including ZIKV, is carried out in membranous replication factories (RFs), which concentrate the viral NS proteins, viral RNA and a number of unidentified host factors [19, 33–34]. Cryo-electron tomography studies of ZIKV and other flavivirus-infected cells revealed the formation of spherical single-membrane vesicles, derived from the endoplasmic reticulum, containing pore-like openings, which are the presumed sites of RNA synthesis [34–37]. Neither the exact protein composition within the flavivirus RFs, nor the stoichiometry of the viral NS proteins is currently known. However, intracellular accumulations of oligomeric polymerases were observed *in vivo*, during viral infections in different representatives of positive (+) and negative (-) ssRNA viruses (e.g. Poliovirus [38], Human Norovirus [39], Sendai virus [40] or Rift Valley Fever virus [41]), indicating the relevance of these quaternary structures in viral replication. In addition, the role of polymerase oligomerization in modulation of the *in vitro* enzyme activity have been extensively studied in several virus families, including flaviviruses, revealing regulatory mechanisms and functions associated to this reversible process [42–46].

The ZIKV NS5 structures reported here show a dimer based helicoidal arrangement of NS5 molecules growing along the crystal c-axes (Fig 6A). Head to tail dimers (Type I interactions) are formed by molecules AB, CD and EF in the P2_1_2_1_2_1_ crystals and by A’B’, C’D’ and E’F’ in the P6_5_ space group (Figs 1A, 2A, 2B and 3). The presence of NS5 dimers in solution has been confirmed by SEC-MALS, AUC and SAXS (Figs 4 and 5). In addition, the mutagenic studies revealed that MTase-MTase interactions and, in particular, those mediated by residues Y25, K28 and K29 are essential for NS5 dimerization (Fig 6B). Dimer-dimer contacts (type II interactions) are established between molecules BC and DE, and B’C’ and D’A”, in the P2_1_2_1_2_1_ and P6_5_ crystals, respectively (Fig 2A and 2C, S4 and S5 Figs). The combination of type I and type II contacts results in the formation of the large helicoidal structures seen in the packing of both crystal forms (Fig 7A) and would be the responsible for the presence of the fibril-like structures observed by both negative staining EM and AFM imaging techniques (Fig 7B and 7C, S9A–S9C, S10A, S10C and S10F Figs). The lack of a regular pattern of the fibril-like structures observed by EM or AFM could be due to the intrinsic interdomain flexibility of the NS5 molecule, as seen in the SAXS experiments (Fig 5). It is also important to remark that these fibril-like structures are not observed in the NS5-Y25A/K28S/K29A variant (S9B and S10D Figs). Hence, the structural and biophysical data presented here shows that the ZIKV enzyme has the ability to form stable dimers as the building blocks for the formation of higher-order oligomers that would participate in the fine-tuned regulation of the multiple enzyme functions in the replication complex. [i.e. (+) and (-) strand RNA synthesis (RdRP domain) and addition of a guanine cap to the 5’ end of (+) strand RNA, followed by two methylation reactions to form a type 1 cap structure at the 5´ end of the nascent RNA (MTase domain)]. These fundamental processes must be precisely regulated to satisfy the virus replication requirements.

**Fig 9:**
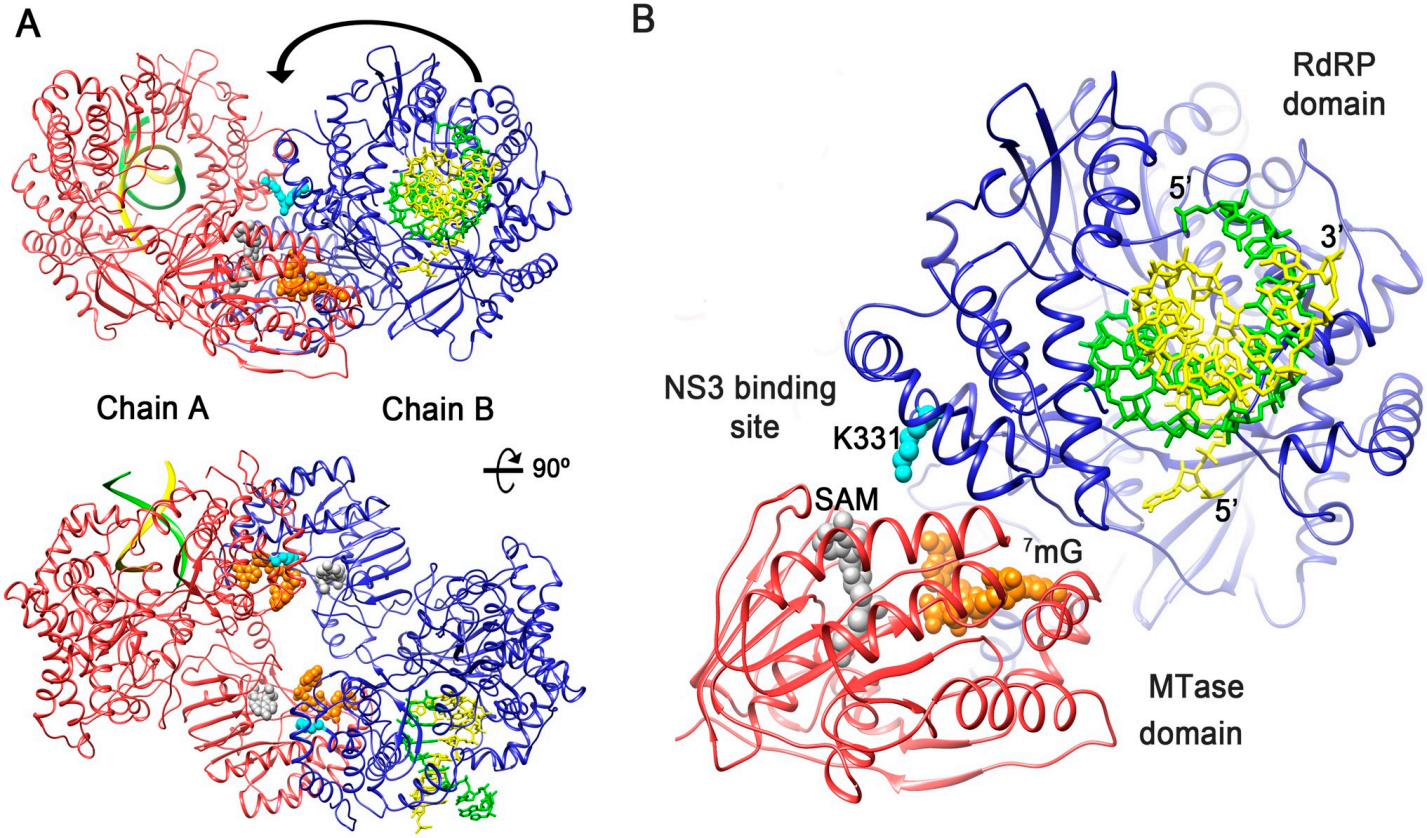
ZIKV NS5 dimers provide a platform for cooperation between RNA synthesis, NS3 binding and 5’-RNA capping. **(A)** Cartoon representation of two views of the NS5 dimer with monomer A shown in red and monomer B in blue. A RNA template/primer is modeled in the polymerase central cavity of the two RdRPs, based in the structure of a PV replication-elongation complex (PDB: 3OL6), with the template strand shown in yellow and primer strand in green. The SAM cofactor (white spheres) and the ^7^mG molecule (orange spheres) have been modeled in the MTase active site by superimposition of the DENV MTase structure (PDB: 3EVE). The RdRP domain residue K331, essential for NS3 binding is shown as spheres in cyan. **(B)** Close up view highlighting the direct connection between the dsRNA exit of one monomer (blue with RNA in yellow and green), the NS3 binding site (cyan spheres) and the entrance to the MTase active site of its partner (red) in the dimer.

Additionally, oligomeric arrangements may mimic the association of the enzyme with other viral and/or host factors in the RFs. For instance, NS3 another player of the flavivirus replication complex has a helicase domain that unwinds the dsRNA and removes the γ-phosphate at the 5′-RNA prior to RNA cap addition. It is expected that individual activities of NS5 and NS3 would be modulated by mutual interactions. Their respective binding sites map to the C-terminal domain of NS3 (residues 566–595) [47] and a region in the RdRP fingertips, containing the conserved charged residue K331 that appears to play a critical role [48–49]. In the ZIKV NS5 dimeric form the NS3 binding sites in the RdRP domain is also in close proximity of the neighbor MTase, facilitating the combined activities (Fig 9).

Looking only at the overall structure of the NS5 monomers, the active sites of the MTase and RdRP domains lay on opposite faces and do not interact with each other (Fig 1B). A similar arrangement was found in the structures of JEV and DENV NS5 monomers (S2 Fig), and large conformational changes were predicted to occur in order to facilitate the MTase to occupy a position, near the exit of the recently synthesized dsRNA and enable 5′-RNA cap transfer [15]. The strictly conserved GTR motif at the N-terminus of the MTase-RdRP linker was proposed to act as a hinge, allowing the required interdomain movements [15, 50].

An alternative hypothesis, implying the coordination of the MTase and RdRP activities, across NS5 molecules in a dimeric form, was previously proposed in light of the crystal structure of DENV NS5 [19]. In the DENV model, the RdRP dsRNA exit site in one monomer and the MTase active site of its partner were oriented in the same direction, allowing coordination between MTase and RdRP domains across NS5 molecules, without the required conformational changes associated to the monomer [19]. Similar to that reported for DENV, in the ZIKV NS5 head-to-tail dimers (Type I interactions; Figs 2A, 2B and 3) the dsRNA exit site of one protomer faces the MTase active site of the adjacent protomer in the dimer (Fig 9A and 9B). As mentioned above, this neighbor MTase is in closer proximity to the NS3 binding site in the partner RdRP (14 Å) than in its own polymerase domain (30 Å), supporting the idea that the different combined activities (dsRNA product unwinding, removal of the 5′-RNA γ-phosphate and cap addition) could be carried out in trans (Fig 9B).

Our *in vitro* primer extension assays show that the NS5-Y25A/K28S/K29A mutant, displaying impaired dimerization activity, is more efficient in primer extension than the wild type enzyme, in particular at high protein concentrations when dimerization is favored, indicating a negative regulatory effect of dimers in chain elongation. These results prompt to speculate that initiation of RNA synthesis and chain elongation processes in flaviviruses could be regulated by a monomer-dimer equilibrium of NS5 so that the monomeric form of the enzyme would favor elongation.

Furthermore, the basic MTase residues K28 and K29 essential for dimer formation are also important for GTP binding [16, 31] and our competition assays show that NS5 oligomerization was hindered at high GTP concentrations (Fig 8B and S11 Fig). Therefore, the direct competition between NS5 oligomerization and GTP binding appears as a possible mode to regulate the cap addition process.

In conclusion, the ZIKV NS5 quaternary arrangement found in this work sheds new light on how the flavivirus multi-task proteins NS5 might coordinate multiple reactions within a single polypeptide, suggesting alternative routes for the discovery of new antivirals, targeting the intermolecular interfaces involved in dimer/oligomer formation.

## Materials and methods

### Production and purification of the recombinant full-length wild type ZIKV NS5 protein

The gene segment covering the coding sequence of ZIKV NS5 (GenBank accession number KU312312.1), containing an additional 6xHis C-terminal tag with and a GSSS spacer optimized for protein expression in *E*. *coli*, was purchased from GenArt (ThermoFisher), including flanking sequences which provide NdeI and EcoRI restriction sites in the 5’ and 3’ ends, respectively.

The ZIKV NS5 gene was cloned in a pMA plasmid vector, pMA/ ZIKV NS5-His. pMA/ ZIKV NS5-His was digested with NdeI and EcoRI FastDigest (ThermoFisher); the fragment containing (2,748bp) the ZIKV NS5 ORF was purified from an agarose gel (NucleoSpin gel extraction kit, Macherey-Nagel) and ligated to pRSET vector (ThermoFisher) previously cleaved with the same enzymes. The resulting plasmid, pRSET ZIKV NS5-His, encodes the ZIKV full-length NS5 fused to a C-terminal 6xHis tag (NS5-His).

*E*. *coli* BL21 (DE3) competent cells were transformed by heat shock (1 pulse of 30 sec at 42°C followed by a 2 min ice incubation step and 1 h at 37°C) with pRSET ZIKV NS5-His and plated on a LB plate containing ampicillin (100 μg/ml). A mix of different BL21 (DE3) colonies were used to inoculate 100 ml of LB medium with 100 μg/ml ampicillin to reach an OD_600_ of 0.6 at 37 °C at 200 rpm as starter culture. Two liters of LB media (100 μg/ml ampicillin) were inoculated with the starter culture and incubated under identical conditions. When the optical density at 600 nm reached 0.6–0.8, isopropyl β-D-1-galactopyranoside was added to the culture at a final concentration of 0.1 mM and incubated at 17 °C for 18 h. Cells were harvested by centrifugation, and washed with 100 ml of phosphate-buffered saline and frozen at -20 °C.

The frozen pellet was thawed on ice and resuspended in lysis buffer (50 mM Tris pH 7.0, 500 mM NaCl, 10% glycerol, 15 mM imidazole, 1 mM DTT) supplemented with protease inhibitors (Complete EDTA-Free; Roche), RNAse (Neo-Biotech) and DNAse I (Bio Basic, Canada Inc.) and 5 mM MgCl_2_ lysed by passage through a cell disrupter at 1.4 Bar (Constant Systems, UK). The extract was centrifuged at 19,000 rpm for 20 min in a JA25.50 rotor (Beckman Coulter). Supernatants were resolved by metal affinity chromatography purification using a nickel-charged HisTrapFF column (GE Healthcare). The column-bound NS5-His was washed with 5 resin volumes of washing buffer (50 mM Tris pH 7.0, 500 mM NaCl, 10% glycerol, 1 mM DTT, 5 mM imidazole) and eluted with a linear gradient to 500 mM imidazole in 50 mM Tris pH 7.0, 500 mM NaCl, 10% glycerol, 1 mM DTT. Fractions containing NS5-His were pooled and dialyzed ON at 4 °C against 50 mM Tris pH 7.0, 300 mM NaCl, 10% glycerol, 1 mM DTT. Dialyzed protein was subsequently diluted with 50 mM Tris pH 7.0, 10% glycerol, 1 mM DTT to reduce de NaCl concentration to 100 mM and loaded onto a HiTrap SP HP column and eluted with a linear gradient to 1 M NaCl.

Fractions containing NS5-His were pooled, concentrated and further purified by size-exclusion chromatography on a Superdex 200 HR 10/300 column exchanging the buffer to 50 mM MES [2-(N-morpholino)ethanesulfonic acid] pH 6.0, 500 mM NaCl, 10% glycerol and 5 mM DTT. Finally, fractions containing the purified NS5-His were pooled and concentrated to 10 mg/ml using Amicon Ultra 30K filters (Millipore), flash frozen in liquid nitrogen and stored at -80 °C.

### Production and purification of NS5 mutants

The MTase and RdRP domains of ZIKV NS5 were amplified by PCR from the purchased NS5 ORF, using Fw (5’CGCATATGGGTGGTGGCAC3’) and Rv (5’CGCATATGGGTGGTGGCAC3’) primers for the MTase domain (residue 1–269), and Fw (5’CGCATATGG AAGCACCGAATATG3’) and Rv (5’GCGAATTCCTACAGATAACGAACCTG3’) primers for RdRP domain (residues 271–894), with NdeI and EcoRI site for both Fw and Rv primers, respectively. PCR products obtained with Phusion (Thermo Scientific) were purified, digested with the proper restriction enzymes and cloned into pGEX4T-TEV [a modified version of pGEX-4T-1 expression vector including a spacer region and a TEV protease cleavage site (5’GAT TAC GAT ATC CCA ACG ACC GAA AAC CTG TAT TTT CAG GGC3’) cloned using the BamHI/SmaI sites], with identical enzymes, generating pGEX4T-2TEV-ZIKV-MTase and pGEX4T-2TEV-RdRP plasmids. Mutations Y25A/K28S/K29A in NS5 were introduced by PCR, using the Fw (5’TTACATATGGGTGGTGGCACCGGTGAAACCCTGGGTGAAAAATGGAAAGCACGTCTGAATCAGATGAGCGCACTGGAATTTGCTAGCTACAGTGCAAGC3’) and Rv (5’GCGAATTCCAGAACACCCGG3’) primers with NdeI and EcoRI sites, the fragments were digested and cloned into the pRSET maintaining the 6xHis tag at the C-terminus.

DNA constructs were transformed into BL21 (DE3) *E*. *coli* cells and expressed as the full-length NS5. Recombinant mutant proteins were purified using a GST-trap column (GE Healthcare) equilibrated with lysis buffer and eluted in the same buffer supplemented with 10 mM of reduced glutathione. Eluted fractions were pooled and purified by size-exclusion chromatography on a Superdex 200 HR 10/300 column exchanging the buffer for 50 mM MES [2-(N-morpholino)ethanesulfonic acid] pH 6.0, 500 mM NaCl, 10% glycerol and 5 mM DTT. Fractions containing the proteins of interest were collected and concentrated in Amicon centrifuge filter devices (MW cutoff 10 kDa) to a final concentration of 10 mg/ml and stored at -80 °C.

### Crystallization and data collection

Initial crystallization trials of NS5-His were performed by the sitting-drop vapour diffusion method at both 277, 290 and 293 K in 96-well Greiner plates using a nanolitre-drop crystallization robot (Cartesian) and several screens available at the Automated Crystallography Platform (PAC) (IBMB-CSIC, Barcelona). Protein solution (10 mg/ml in 50 mM MES pH 6.0, 500 mM NaCl, 10% glycerol, 5 mM DTT) droplets of 150 nl were mixed with 150 nl precipitant solution; the volume reservoir was 90 μl. Small bar-like crystals appeared within 1 day in 3 different conditions: a) 0.2M lithium sulphate anhydride, 0.1 M CAPS pH 10.5, 1.2 M sodium dihydrogen phosphate 0.8 M dipotassium hydrogen phosphate, b) 0.1 M sodium acetate pH4.5, 0.8 M sodium dihydrogen phosphate, 1.2 M dipotassium hydrogen phosphate and c) 1.8 M sodium/potassium phosphate, pH 6.9. After optimization in 96-well Greiner plates, rectangular and hexagonal based prism crystals were obtained using the sitting-drop technique by mixing 300 nl of protein with an equal volume of the reservoir solution (0.1 M sodium acetate pH 4.5–5.4, 0.8–1.15 M sodium dihydrogen phosphate, 0.9–1.2 M dipotassium hydrogen phosphate) equilibrated against a 90 μl reservoir. Prior to data collection, crystals were harvested in cryo-loops (Hampton Research), soaked for 1 min in a reservoir solution and 20% (v/v) glycerol, and flash-frozen in liquid nitrogen.

Given that the first diffraction data obtained were at low resolution, crystal growth conditions and freezing were optimized. For this purpose, different salt concentrations in harvesting buffer (additional precipitant concentration, between 5 to 20%) as well as different types of cryoprotectant agents (glycerol, ethylene glycol, paraffin, mineral oil, silicon and sugars) were studied. Even crosslinking of crystals by vapour diffusion with 2% (v/v) of glutaraldehyde at different times were assayed without success. After optimization, the best diffracting crystals were harvested in a cryo buffer containing 5% higher concentration of phosphate salts than in the crystallization condition and 25% of glycerol (for orthorhombic crystals) or a mix of 4% sucrose, 2% glucose, 10% glycerol and 8% ethylene glycol in addition to the 5% of higher salt concentration (for hexagonal crystals).

Diffraction intensities of the orthorhombic crystals were collected on ID23-1, ID23-2 and ID30A-3 beamlines at the European Synchrotron Radiation Facility (ESRF, Grenoble, France) and the XALOC beamline at the ALBA Synchrotron (Cerdanyola del Valles, Spain) for the hexagonal crystals. All data sets were collected at 100 K. Diffraction data were processed using XDS [51].

All datasets collected from orthorhombic crystals were pairwise-compared with SCALEIT (CCP4 suite) [52–53], in a systematic manner, using a home-made script. Eleven partial datasets from different crystals were subsequently merged with XSCALE [54]. Space group determination and data reduction were performed with POINTLESS/AIMLESS [55]. This processing resulted in a complete data set at 5 Å resolution (S1 Table) that was used to solve the structure of NS5 by molecular replacement (see below). A subsequent inspection of the data using the STARANISO software [56], currently available in the web server (http://staraniso.globalphasing.org), revealed that these crystals were highly anisotropic with diffraction limits of ellipsoid fitted to diffraction cut-off surface of: 5.85, 7.44, and 3.76 Å along the a*, b*, and c* axes, respectively. STARANISO analyses the anisotropy decay of the mean intensities (I), constrained by the crystal symmetry, modifies the TRUNCATE procedure and analyses the decay of the local average of I/σ(I) in different directions, providing the basis for an anisotropic resolution cut-off. The criteria used in determination of the resolution cut-off was: Rpim = 0.6, I/σI = 2.0 and CC1/2 = 0.3. When the anisotropy correction was applied to the unmerged data after cut-off produced a best resolution limit of 3.98 Å in direction 0.071 a* + 0.010 b* + 0.997 c* and a lowest cut-off limit of 7.39 Å in direction 0.857 b*+0.514c* (S1 Table).

The diffraction intensities from a single hexagonal crystal were scaled and merged using SCALA [54], resulting in a complete data set at 4.8 Å (S1 Table).

### Structure determination

Full-length NS5 protein crystallized in two different space groups: hexagonal P6_5_ and orthorhombic P2_1_2_1_2_1_, with unit cell parameters of a = b = 234.58 Å, c = 406.12 Å and, a = 191.06 Å, b = 192.06 Å, c = 407.23 Å, with six independent molecules in each crystal asymmetric unit (Matthews coefficient (Vm) of 5.38 and 6.32 corresponding to a solvent content of 77% and 80% for the orthorhombic and hexagonal space groups, respectively).

The P2_1_2_1_2_1_ structure was solved by a combination of molecular replacement with PHASER [57–58], using two independent search models derived from the ZIKV MTase (PDB: 5KQR) and JEV RdRP (PDB 4K6M) domains, and manual fitting after density modification with SHELXE [59], starting from the coordinates of the partial solution yielded a map where the missing domains could be manually fitted [60]. A global NCS was used during the initial refinement with Refmac5 [61], owing to the presence of six copies of the NS5 monomer in the crystal asymmetric unit. Subsequent refinement rounds were performed by Phenix [62], using the 4 Å data corrected by STARANISO and the NCS was relaxed to a torsional NCS with groups automatically defined by the program. This refinement with Phenix was alternated with manual model rebuilding performed with program Coot [63]. The P6_5_ structure was solved by molecular replacement with PHASER, using one entire ZIKV NS5 monomer from the orthorhombic structure as a search model. Refinement was done alternating cycles of refinement in Refmac5 [61], applying torsional NCS and secondary structure restraints, with manual rebuilding using Coot [63]. Data refinement statistics are listed in S1 Table. Superimpositions of structures were carried out using Coot. Calculation of contact surfaces were performed with PISA [64]. Illustrations were prepared with Chimera [65].

### Molecular weight calculations

For analysis of the molecular weight of the monomeric and dimeric forms of ZIKV NS5 in solution, SEC combined with light scattering analysis was used. The SEC data were measured using a Superdex 200 10/300 GL column (GE Healthcare; MW range: 10–600 kDa), connected to high-performance liquid chromatography system (Shimadzu), equipped with an autosampler. The elution from SEC was monitored by a UV detector, differential refractometer (OPTI-rEx, Wyatt Corp.) and static, multiangle laser LS detector (DAWN-HELEOS, Wyatt Corp.). The SEC-UV/MALS/RI system was equilibrated in 50 mM MES pH 6.0, 500 mM NaCl, 10% glycerol, 5 mM DTT or 50 mM HEPES pH 7.0, 500 mM NaCl, 10% glycerol, 5 mM DTT at the flow rate of 0.5ml/min. The ASTRA 7 software (Wyatt Corp.) was used for data collection and analysis.

### Analytical ultracentrifugation

AUC experiments were performed at 20 °C in a Beckman Optima XL-I, operating under velocity sedimentation (SV) mode using an AN-50 Ti rotor with two-channel charcoal-filled centerpieces. Prior to the measurements, ZIKV NS5 buffer was exchanged to 50 mM HEPES pH 7.0, 500 mM NaCl, 5% glycerol, 0.4 mM DTT using Amicon Ultra 30K filters (Millipore). Sedimentation velocity experiments were performed at 43,000 rpm loading 0.5 mg/ml protein. Data were collected at 280 nm in a continuous mode and fitted with SEDFIT [66].

### BS3 cross-linking experiments

Crosslinking experiments were performed in 20 mM HEPES pH 7.0, 150 mM NaCl using 5 μM of NS5 protein in presence of 10 μM of BS3 (bis(sulfosuccinimidyl)suberate, ThermoFisher Scientific) in 100μL. When indicated, GTP was added to 50 mM final concentration. Reactions were incubated 45 min at 4 °C and quenched with 50 mM Tris buffer. Crosslinked samples were resolved on 7.5% SDS-polyacrylamide gels stained with coomassie blue.

### Small-angle X-ray scattering

Samples were prepared as described in the protein production and purification section and analyzed in fresh storage buffer (50 mM MES [2-(N-morpholino)ethanesulfonic acid] pH 6.0, 500 mM NaCl, 10% glycerol and 5 mM DTT). Scattering data were measured at the BioSAXS beamline (BM29, ESRF) over a range of concentrations (40 μl sample 0.5–6 mg/ml) to evaluate particle interaction effects. Sample measurements were collected for 10 frames of 5s exposure flanked by two exposures to buffer samples (SEC buffer) and all experiments were performed at 10 °C. Scattering curves were calculated using the ATSAS package [67], by subtracting the scattering of the buffer. Data quality was assessed for aggregation using Guinier plots, and Kratky plots were used to assess protein folding. The radius of gyration (Rg), experimental maximum particle diameter (Dmax) and molecular weight (MW) were calculated in Primus and the experimental molecular weight was estimated using the Porod Volume [68]. *Ab initio* models were generated using GASBOR [69]. *Ab initio* models for the scattering curve at 6 mg/ml were generated imposing a P2 symmetry. We used the ensemble optimization method (EOM) to determine the conformational flexibility of the NS5 monomer [27–28]. An ensemble of 10,000 random conformations was generated by keeping the RdRP domain constant and allowing the MTase domains (residues 1–268) to adopt any random conformation. EOM selected the optimized sub-ensemble from the random pool.

### Transmission electron microscopy (TEM)

4 μl of a purified protein sample (2 μM) in buffer 50 mM MES pH 6.0, 50 mM NaCl, 5 mM DTT were placed on carbon-coated copper grids and blotted after 1 minute. Alternatively, protein samples were diluted in 50 mM MES pH 6.0, 250 mM NaCl, 5 mM DTT and placed on copper collodion-coated grids covered with 3 nm carbon layer. In both cases, 15s of 25 mA glow discharge was applied to grids before protein binding and blotting after 1 minute.The samples were then stained with 2% (w/v) uranyl acetate, dried and observed at calibrated magnifications of 150,000x and 200,000x using a Jeol JEM 1010 100 kV transmission electron microscope (TEM) equipped with a Megaview 1kx1k CCD digital camera.

### Atomic force microscopy

Protein samples (10 mg/ml) were diluted until 10 μg/ml in protein storage buffer (50 mM MES pH 6.0, 500 mM NaCl, 10% glycerol, 5 mM DTT) and 20 μl immediately deposited onto freshly cleaved muscovite mica. After 10 min at room temperature, the mica surface was rinsed three times with 300 μl of protein buffer to remove molecules that were not firmly attached. Surfaces were scanned and images acquired by tapping-mode in liquid with an AFM CYPHER S (Asylum Research, Oxford Instruments) instrument from the Scientific and technical platform Partnership for Soft Condensed Matter (PSCM, ESRF Grenoble) using a SNL-C cantilever (Bruker). Cantilever oscillation frequency was tuned to the resonance frequency of ~12.7 kHz and images of 501.96 nm x 501.96 nm were obtained at a scanning frequency of 2.5 Hz. Images were processed by Gwyddion 2.48 software [70].

### Polymerase activity assays

The polymerase elongation activity was measured using a nonradioactive coupled-enzyme assay described previously [29]. Briefly, 1 μM of fluorescently labeled RNA primer Cy5.5–5’AGAACCUGUUGAACAAAAGC3’ and 1 μM unlabeled RNA template 5’AUUAUUCGGCUUUUGUUCAACAGGUUCU3’ (IDT, Illinois, USA) template were mixed in 50 mM NaCl in deionized RNase-free water, incubated at 95°C for 10 min, and then slowly cooled to room temperature. The primer extension assays were performed in a 20 μl reaction mixture containing reaction buffer (5 mM Tris-HCl, pH 7.5, 10 mM DTT, 5 mM MnCl_2_, 0.5% Triton X-100, 0.01 U RNasin, 10% glycerol), 10 nM primer/template complex, and decreasing concentrations (900, 180, 18 and 9 nM) of the NS5 proteins (wild type and NS5- Y25A/K28S/K29A mutant). The reaction was initiated by the addition of rNTPs at a final concentration of 100 μM, followed by incubation for 1 h at 35°C. The reactions were quenched by the addition of 40 μl quenching buffer (8 M urea, 90 mM Tris base, 29 mM taurine, 10 mM EDTA, 0.02% SDS, 0.1% bromophenol blue). The samples were then denatured at 95 °C for 15 min, and the primer extension products were resolved on 15% denaturing polyacrylamide gels (urea-PAGE) using 1X TBE buffer (100 mM Tris base, 100 mM Boric acid, 2 mM EDTA). Gels were scanned using an Odyssey infrared imaging system. The images were analyzed using Fiji software.

## Supporting information

S1 Fig Electron density maps of the ZIKV NS5 structure in the P2_1_2_1_2_1_ space group. **(A)** 2Fo-Fc electron density map (1.5σ, cyan) at 4Å resolution after refinement, covering the main chain of the protein in ZIKV NS5 dimer represented in ribbons (Chain E, yellow; chain F, magenta) with the putative SAM/SAH molecules depicted in sticks. **(B-C)** Comparative close up of a MTase region shown with the corresponding 2Fo-Fc electron density maps at 5Å (left) and 4 Å resolution, with anisotropy correction (right).
(TIF)

S2 Fig Inter-domain flexibility in NS5. **(A)** Superposition of the RdRP domains of ZIKV NS5. The coordinates of three different molecules are superimposed, chains A (blue) and E (cyan) in the P2_1_2_1_2_1_ crystal structure (PDB: 5M2X; this work) and chain A (grey) of the P2_1_2_1_2 structure (PDB: 5FTR) [20], showing the relative movements of the MTase domains. **(B)** Superposition of the RdRP domains of ZIKV, chain A (blue) with JEV NS5 (PDB: 4K6M; chain A, orange) [17]. **(C)** Structural alignment between the MTases domains of ZIKV NS5 (blue) and Dengue 3 NS5 (PDB: 5CCV, chain A, red) [19].
(TIF)

S3 Fig Sequence alignment of full length flavivirus NS5 proteins of known structure. Alignments would correspond to the Suriname ZIKV human isolate (PDB: 5M2X, 5M2Z described in this work), ZIKV strain MR776 (PDB: 5FTR) JEV (PDB: 4K6M) and DENV (PDB: 5CCV). White letters in red boxes indicate identity, and red letters residue conservation.
(TIF)

S4 Fig Contact surfaces in ZIKV NS5 P6_5_ crystal structure. Schematic representation of the asymmetric unit in the P6_5_ crystals, showing the disposition and the interfaces (I and II) between chains A’ to F’ (left panel). The orientation of the dsRNA exit channels in the RdRP domains are indicated with arrows. Measurements of the buried surfaces between NS5 molecules forming dimers (I) or between two adjacent dimers (II) (right panel).
(TIF)

S5 Fig Contact interactions along the crystallographic c axis in the P2_1_2_1_2_1_ crystals. **(A)** Organization of the 3 dimers of the asymmetric unit to form long fibers along the unit cell axis c. Panels **B** and **C** panels show the contact interfaces.
(TIF)

S6 Fig Sedimentation velocity analysis of ZIKV NS5. Raw sedimentation profile of absorbance at 280 nm (color circles) acquired versus cell radius and best-fit c(s) model (color lines), acquired at different time points. The bottom panel shows the overlay of the residuals of the fit supplied by SEDFIT software.
(TIF)

S7 Fig SAXS analysis of the ZIKV NS5 protein. Guinier regions **(A)**, Kratky plots **(B)** and Pair-Distance Distributions functions **(C)** for the ZIKV polymerase samples collected at 0.5 mg/ml (top panels) and 6 mg/ml (bottom panels).
(TIF)

S8 Fig MTase-MTase contacts are responsible of NS5 dimer formation. SDS-PAGE 10% of the ZIKV NS5 protein, the left panel shows the full length NS5, the middle panel the MTase domain and the right panel the RdRP domain. 5μM of NS5 protein are crosslinked in presence of 10 μM of BS3 (45 min incubation) in buffer 50 mM MES pH 6.0, 150 mM NaCl, 5 mM DTT. In all panels the first lane shows the molecular weight markers (MWM); second lane: protein/domain control without BS3; third lane: crosslinked protein/domain. The formation of dimers (arrow) and other high order oligomers is evident in the left and middle panels.
(TIF)

S9 Fig Negative staining EM study of NS5 wild type and the NS5-Y25A/K28S/K29A variant. **(A)** Selected area from an electron micrograph of a negative stained sample (2% uranyl acetate) showing the wild type NS5 protein in buffer 50 mM MES pH 6.0, 250 mM NaCl, 5 mM DTT. **(B)** Selected area of the micrograph shown in **A** at higher magnification. **(C)** Selected area from an electron micrograph showing wild type NS5 protein in the same buffer as in **A** at a dilution 1/200. **(D)** Electron micrograph showing the NS5-Y25A/K28S/K29A variant in the same buffer conditions and same concentration as the wild type protein, shown in **A**.
(TIF)

S10 Fig AFM analysis of NS5 WT and the NS5-Y25A/K28S/K29A mutant. **(A)** AFM images of a region 100μm x 86μm of freshly cleaved mica surface incubated with the WT NS5 forming fibril-like structures at pH6.0 (50 mM MES, 500 mM NaCl, 10% glycerol, 5 mM DTT; left panel) and pH7.0 (50 mM HEPES, 500 mM NaCl, 10% glycerol, 5 mM DTT; right panel). The left lateral scale in nm correspond z-axis describing the height of the scanned surface by an artificial color scale. **(B)** NS5-Y25A/K28S/K29A mutant using the same buffers as in **A**. Close up images of a WT NS5 fibril-like oligomer **(C)** and of the NS5-Y25A/K28S/K29A mutant **(D)** observed at pH7.0. **(E)** Profile sections, showing the dimensions of height and width of three random zones measured in the AFM image shown in panel F. **(F)** AFM image of the wild type NS5 and its 3D representation. **(G)** Ribbon diagram of the NS5 dimer shown in two different views, with the protomers colored in blue and red, respectively. Different dimensions of the dimer height and width are indicated.
(TIF)

S11 Fig Effects of nucleotide binding in NS5 dimerization. SDS-PAGE of the ZIKV NS5 wild type protein (5 μM) crosslinked with 10 μM BS3 (45 min incubation), in buffer containing 20 mM HEPES pH 7.0 and 150 mM NaCl, in absence or presence of GTP, GMP and ATP at 25 mM and 10 mM nucleotide concentration.
(TIF)

S1 Table Data collection and refinement statistics. (DOC)

S2 Table Small angle X-ray scattering parameters. (DOCX)
