# Supplementary figures and images for: Supramolecular arrangement of the full-length Zika virus NS5
Source: PLoS Pathog. 2019 Apr 5;15(4):e1007656. doi: 10.1371/journal.ppat.1007656 (PMC6469808; doi:10.1371/journal.ppat.1007656)

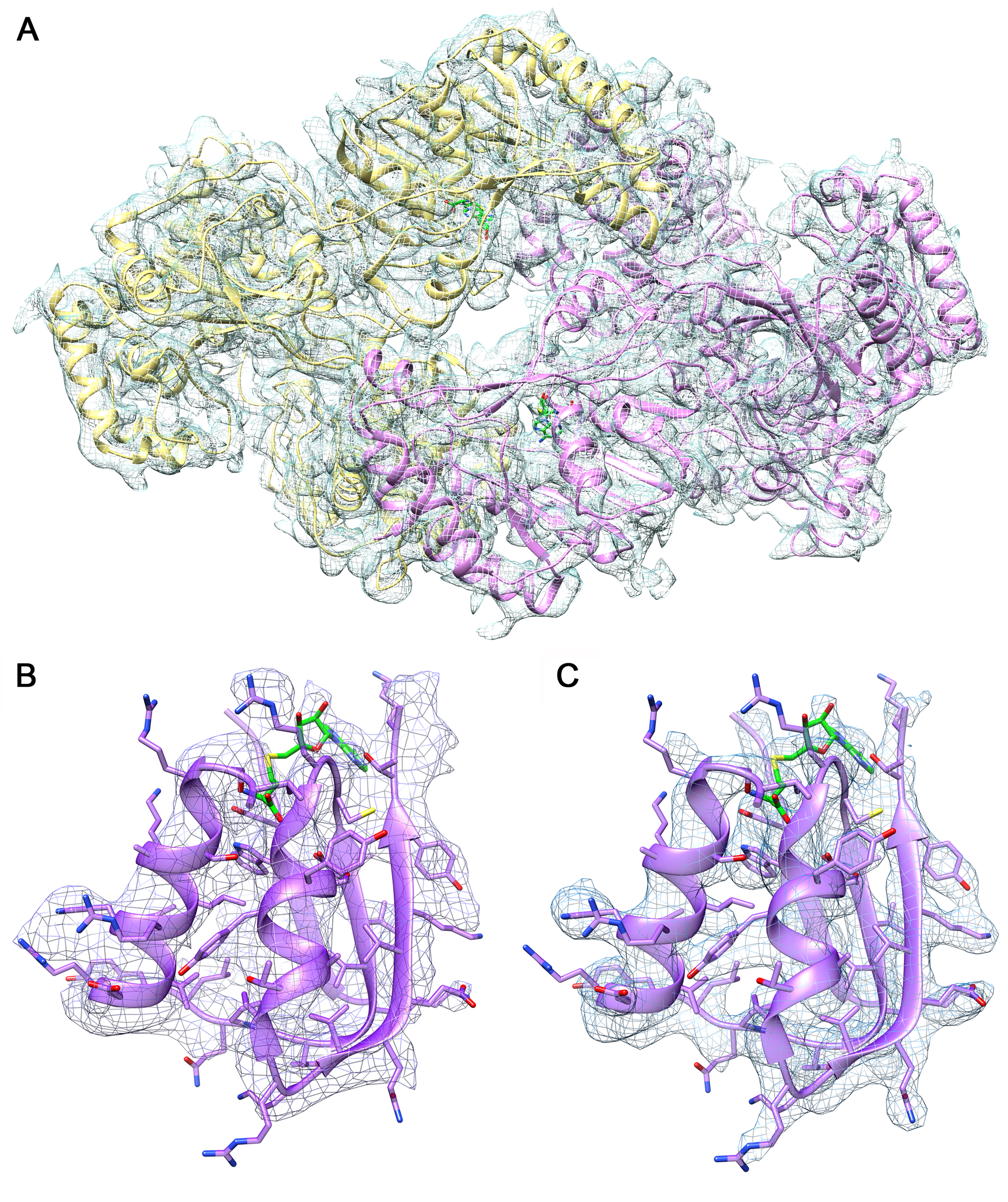

Supplement: S1 Fig — (A) 2Fo-Fc electron density map (1.5σ, cyan) at 4Å resolution after refinement, covering the main chain of the protein in ZIKV NS5 dimer represented in ribbons (Chain E, yellow; chain F, magenta) with the putative SAM/SAH molecules depicted in sticks. (B-C) Comparative close up of a MTase region shown with the corresponding 2Fo-Fc electron density maps at 5Å (left) and 4 Å resolution, with anisotropy correction (right). (TIF) [file ppat.1007656.s001.tif]

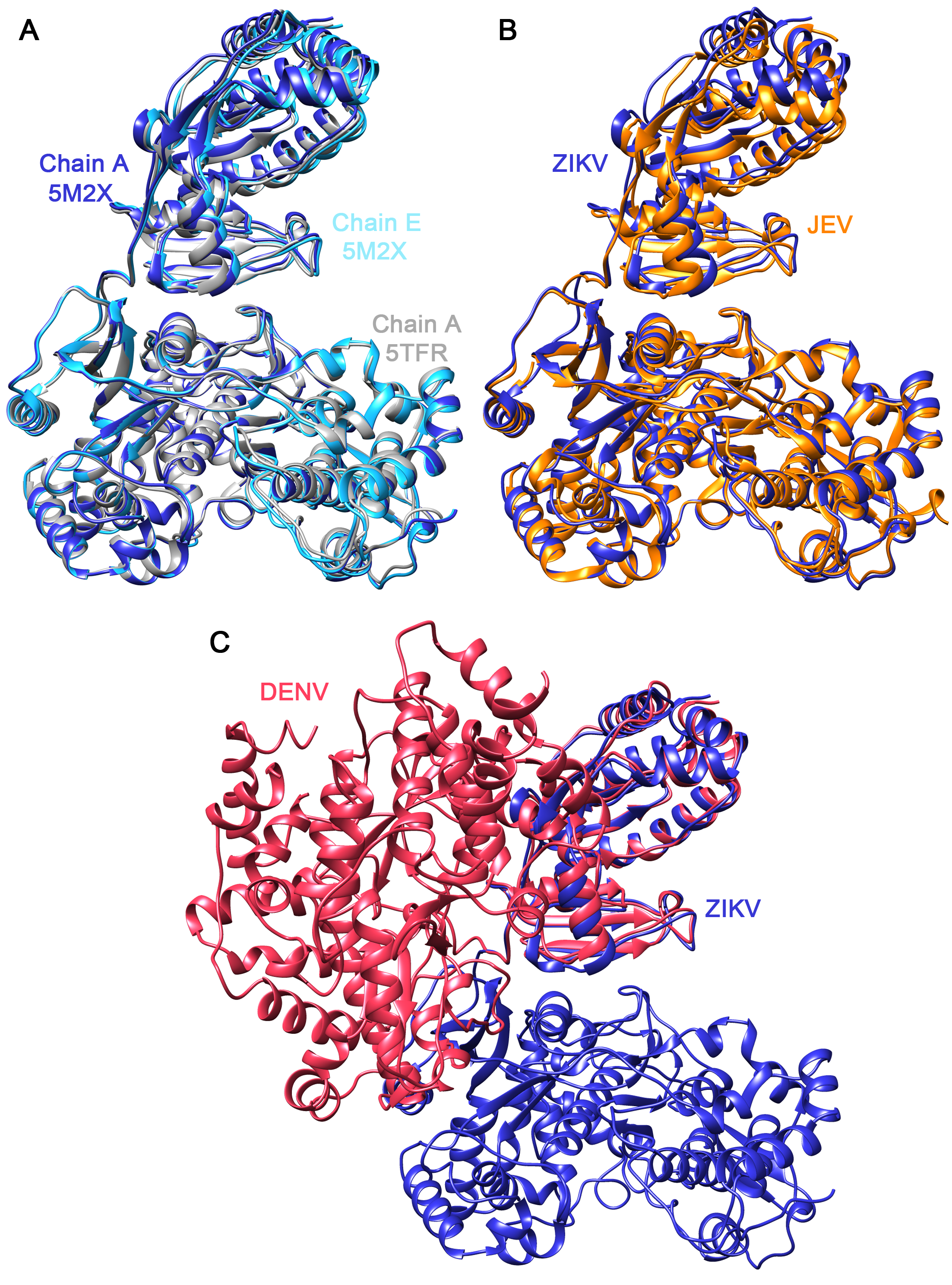

Supplement: S2 Fig — (A) Superposition of the RdRP domains of ZIKV NS5. The coordinates of three different molecules are superimposed, chains A (blue) and E (cyan) in the P212121 crystal structure (PDB: 5M2X; this work) and chain A (grey) of the P21212 structure (PDB: 5FTR) [20], showing the relative movements of the MTase domains. (B) Superposition of the RdRP domains of ZIKV, chain A (blue) with JEV NS5 (PDB: 4K6M; chain A, orange) [17]. (C) Structural alignment between the MTases domains of ZIKV NS5 (blue) and Dengue 3 NS5 (PDB: 5CCV, chain A, red) [19]. (TIF) [file ppat.1007656.s002.tif]

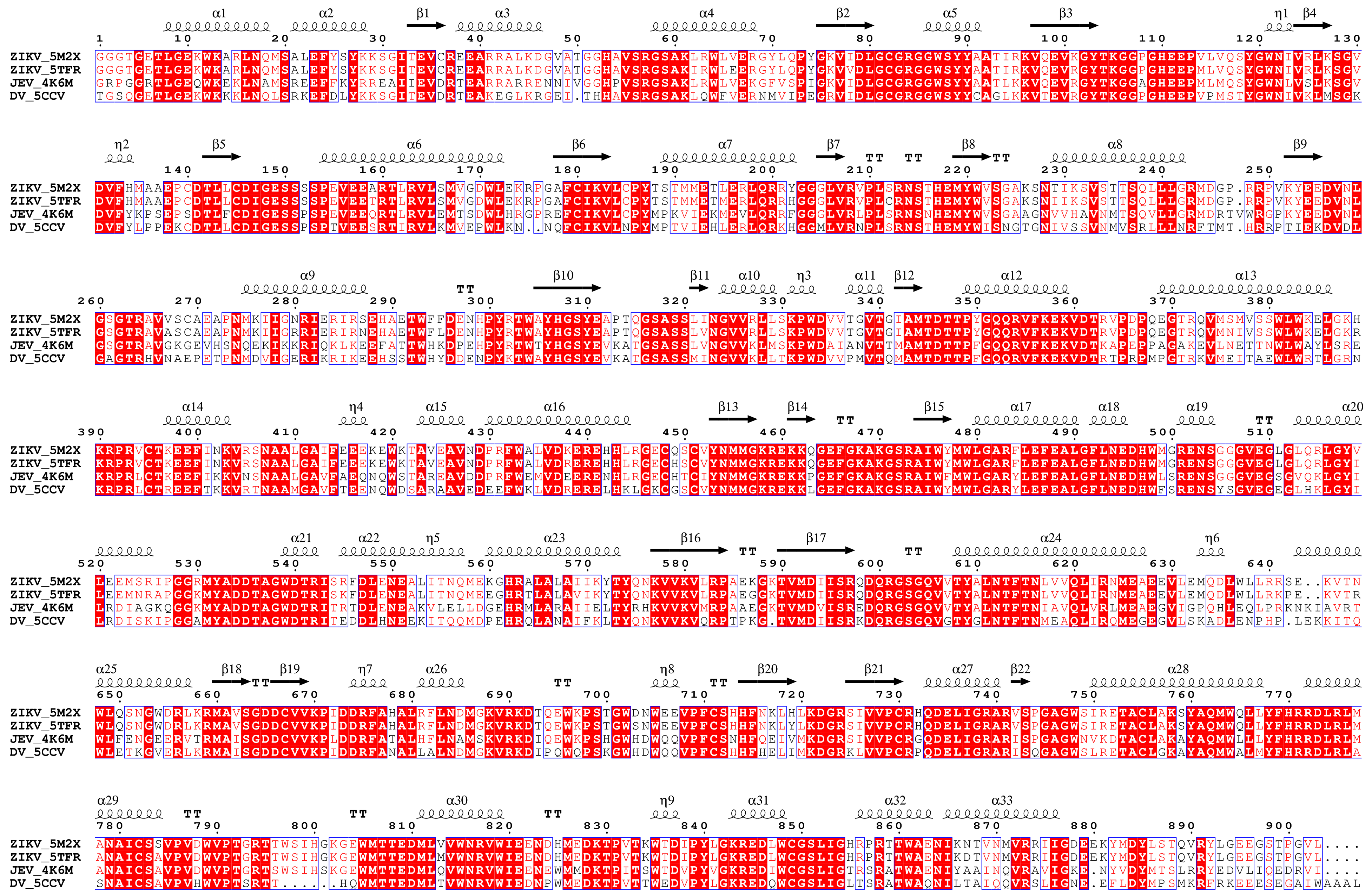

Supplement: S3 Fig — Alignments would correspond to the Suriname ZIKV human isolate (PDB: 5M2X, 5M2Z described in this work), ZIKV strain MR776 (PDB: 5FTR) JEV (PDB: 4K6M) and DENV (PDB: 5CCV). White letters in red boxes indicate identity, and red letters residue conservation. (TIF) [file ppat.1007656.s003.tif]

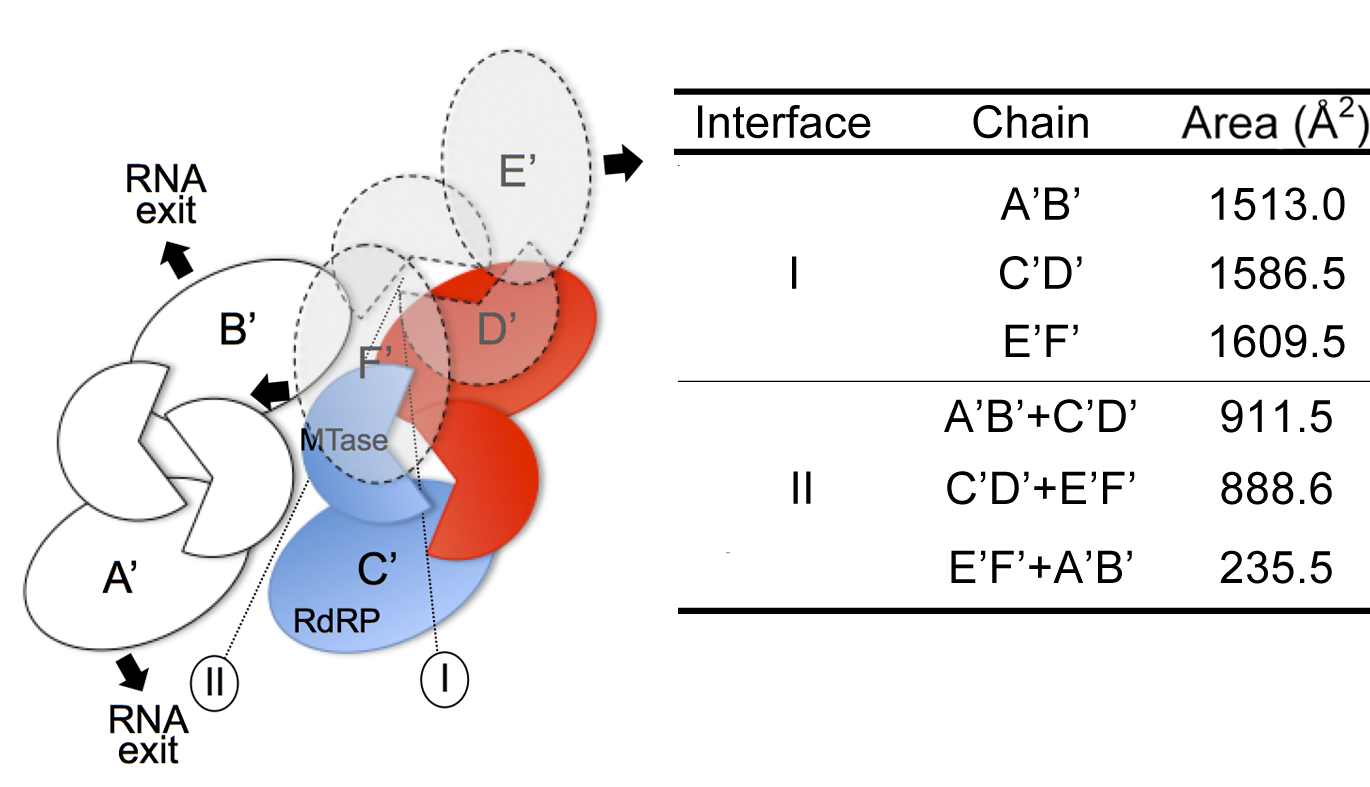

Supplement: S4 Fig — Schematic representation of the asymmetric unit in the P65 crystals, showing the disposition and the interfaces (I and II) between chains A’ to F’ (left panel). The orientation of the dsRNA exit channels in the RdRP domains are indicated with arrows. Measurements of the buried surfaces between NS5 molecules forming dimers (I) or between two adjacent dimers (II) (right panel). (TIF) [file ppat.1007656.s004.tif]

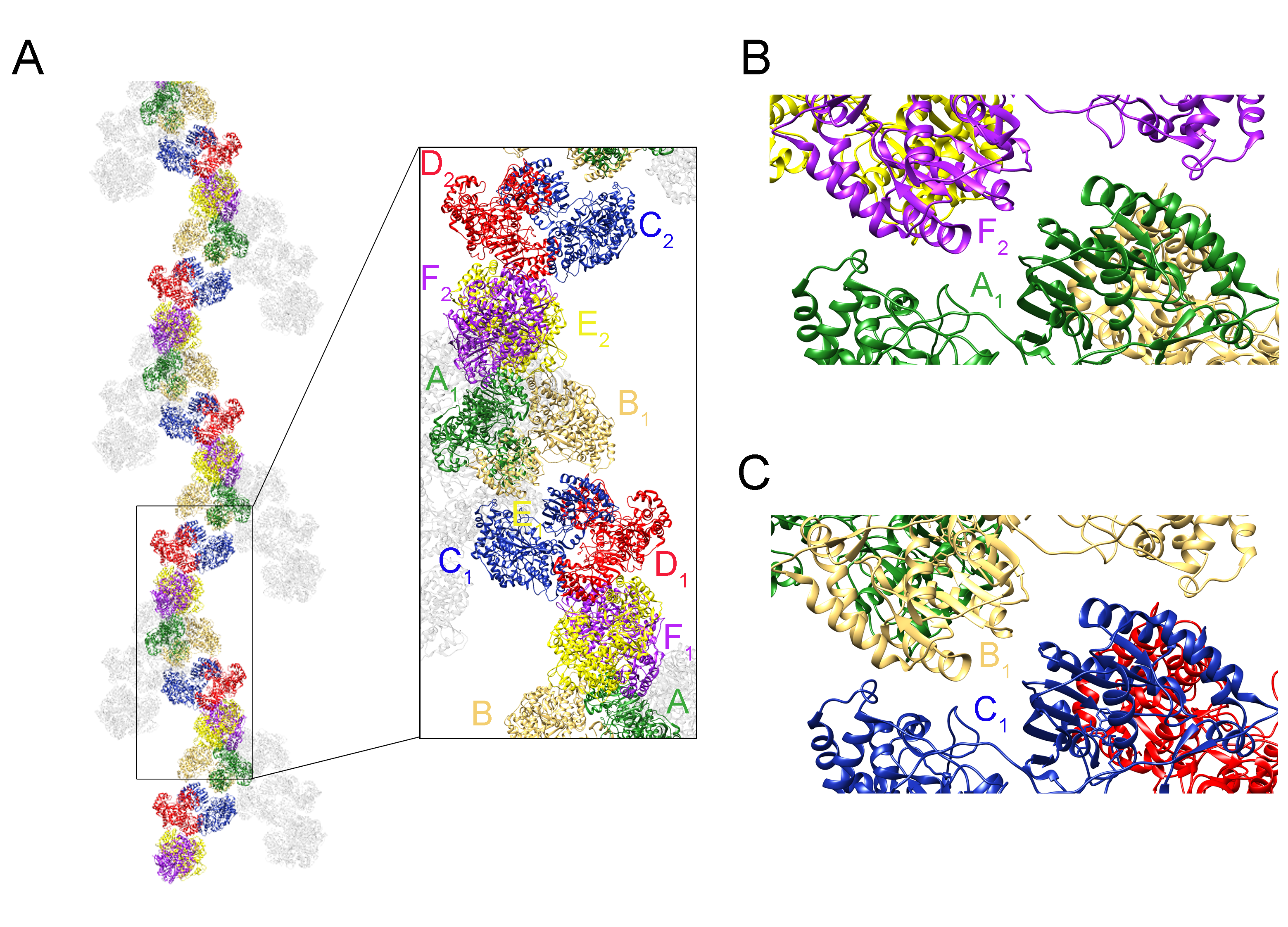

Supplement: S5 Fig — (A) Organization of the 3 dimers of the asymmetric unit to form long fibers along the unit cell axis c. Panels B and C panels show the contact interfaces. (TIF) [file ppat.1007656.s005.tif]

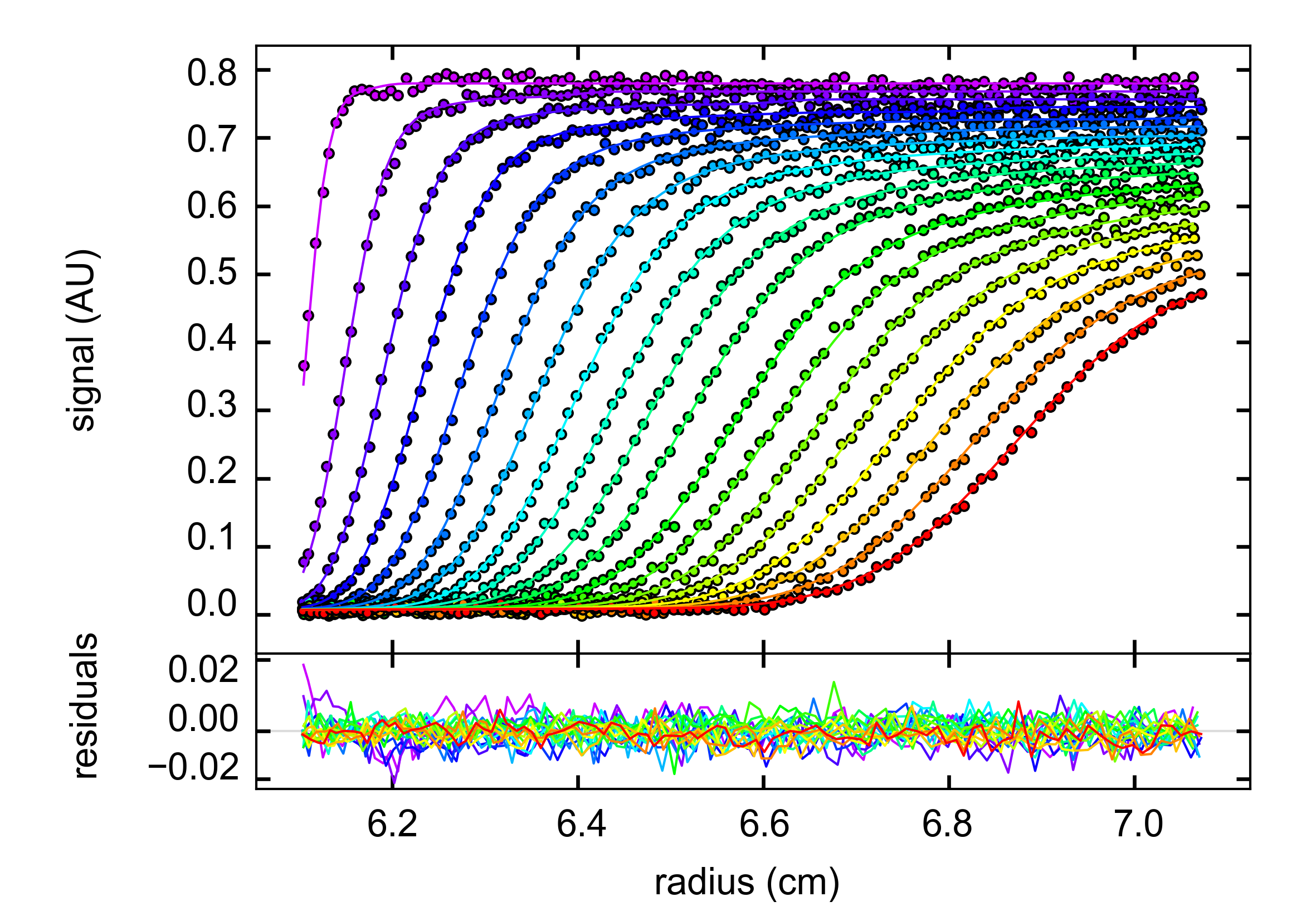

Supplement: S6 Fig — Raw sedimentation profile of absorbance at 280 nm (color circles) acquired versus cell radius and best-fit c(s) model (color lines), acquired at different time points. The bottom panel shows the overlay of the residuals of the fit supplied by SEDFIT software. (TIF) [file ppat.1007656.s006.tif]

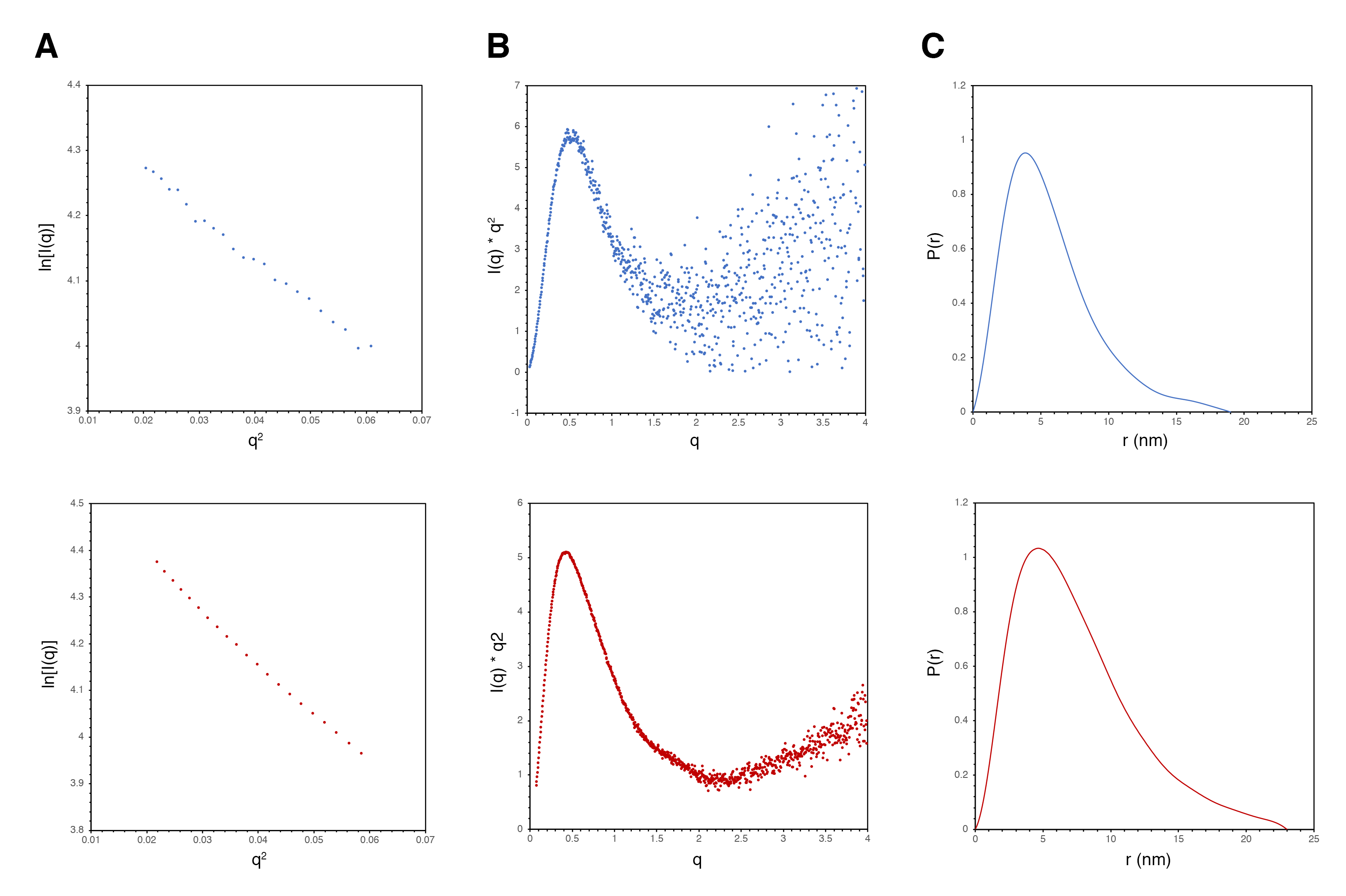

Supplement: S7 Fig — Guinier regions (A), Kratky plots (B) and Pair-Distance Distributions functions (C) for the ZIKV polymerase samples collected at 0.5 mg/ml (top panels) and 6 mg/ml (bottom panels). (TIF) [file ppat.1007656.s007.tif]

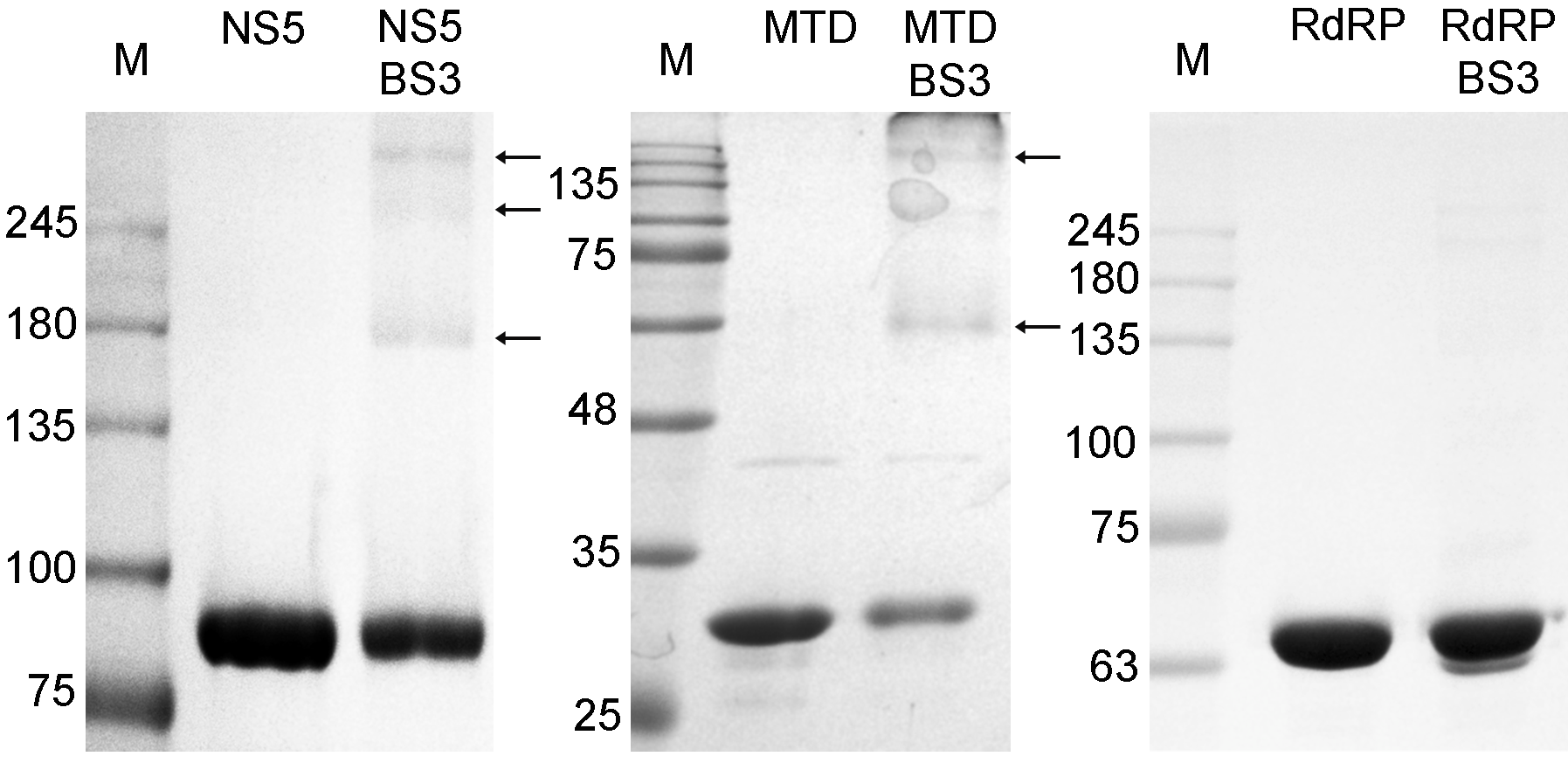

Supplement: S8 Fig — SDS-PAGE 10% of the ZIKV NS5 protein, the left panel shows the full length NS5, the middle panel the MTase domain and the right panel the RdRP domain. 5μM of NS5 protein are crosslinked in presence of 10 μM of BS3 (45 min incubation) in buffer 50 mM MES pH 6.0, 150 mM NaCl, 5 mM DTT. In all panels the first lane shows the molecular weight markers (MWM); second lane: protein/domain control without BS3; third lane: crosslinked protein/domain. The formation of dimers (arrow) and other high order oligomers is evident in the left and middle panels. (TIF) [file ppat.1007656.s008.tif]

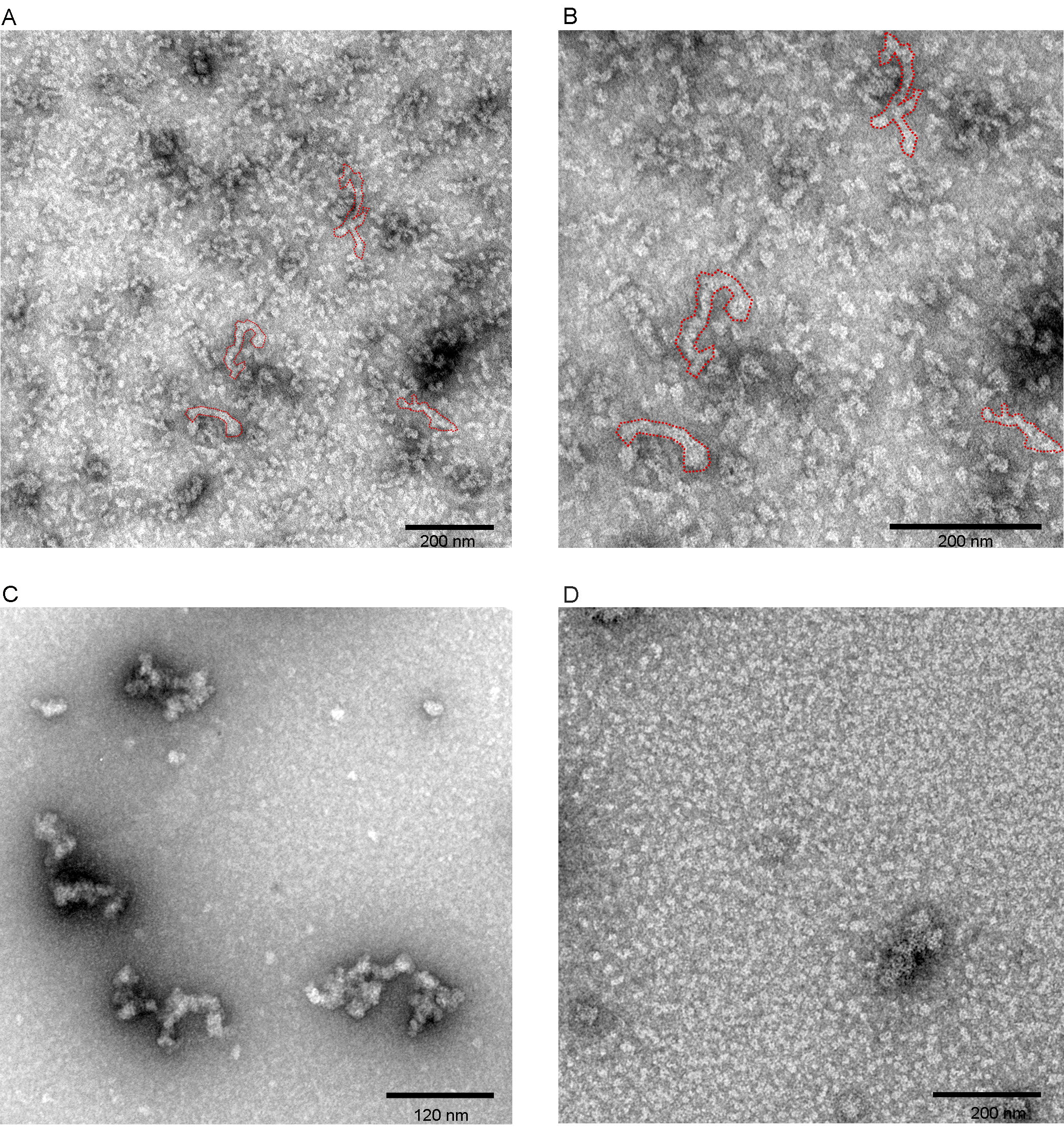

Supplement: S9 Fig — (A) Selected area from an electron micrograph of a negative stained sample (2% uranyl acetate) showing the wild type NS5 protein in buffer 50 mM MES pH 6.0, 250 mM NaCl, 5 mM DTT. (B) Selected area of the micrograph shown in A at higher magnification. (C) Selected area from an electron micrograph showing wild type NS5 protein in the same buffer as in A at a dilution 1/200. (D) Electron micrograph showing the NS5-Y25A/K28S/K29A variant in the same buffer conditions and same concentration as the wild type protein, shown in A. (TIF) [file ppat.1007656.s009.tif]

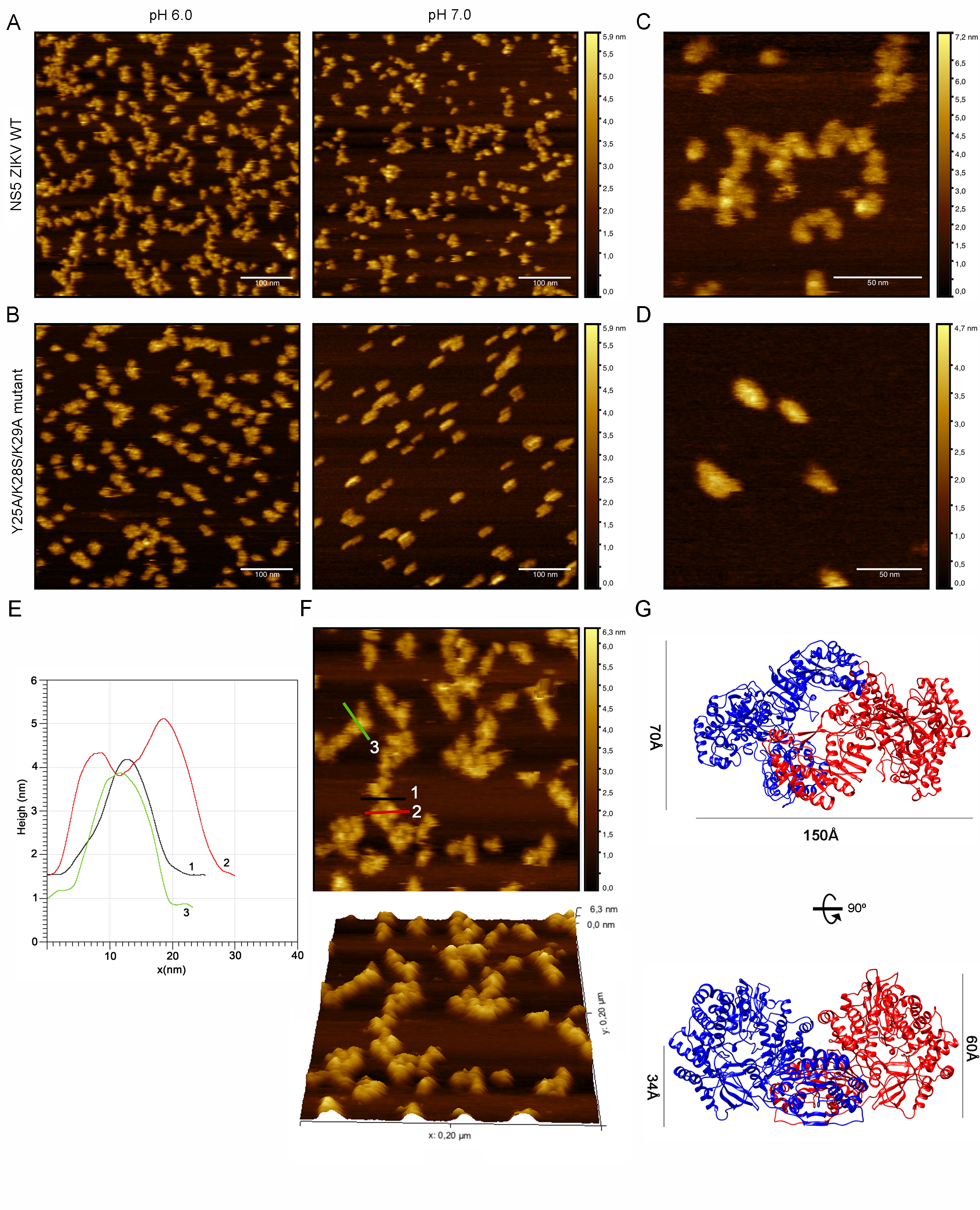

Supplement: S10 Fig — (A) AFM images of a region 100μm x 86μm of freshly cleaved mica surface incubated with the WT NS5 forming fibril-like structures at pH6.0 (50 mM MES, 500 mM NaCl, 10% glycerol, 5 mM DTT; left panel) and pH7.0 (50 mM HEPES, 500 mM NaCl, 10% glycerol, 5 mM DTT; right panel). The left lateral scale in nm correspond z-axis describing the height of the scanned surface by an artificial color scale. (B) NS5-Y25A/K28S/K29A mutant using the same buffers as in A. Close up images of a WT NS5 fibril-like oligomer (C) and of the NS5-Y25A/K28S/K29A mutant (D) observed at pH7.0. (E) Profile sections, showing the dimensions of height and width of three random zones measured in the AFM image shown in panel F. (F) AFM image of the wild type NS5 and its 3D representation. (G) Ribbon diagram of the NS5 dimer shown in two different views, with the protomers colored in blue and red, respectively. Different dimensions of the dimer height and width are indicated. (TIF) [file ppat.1007656.s010.tif]

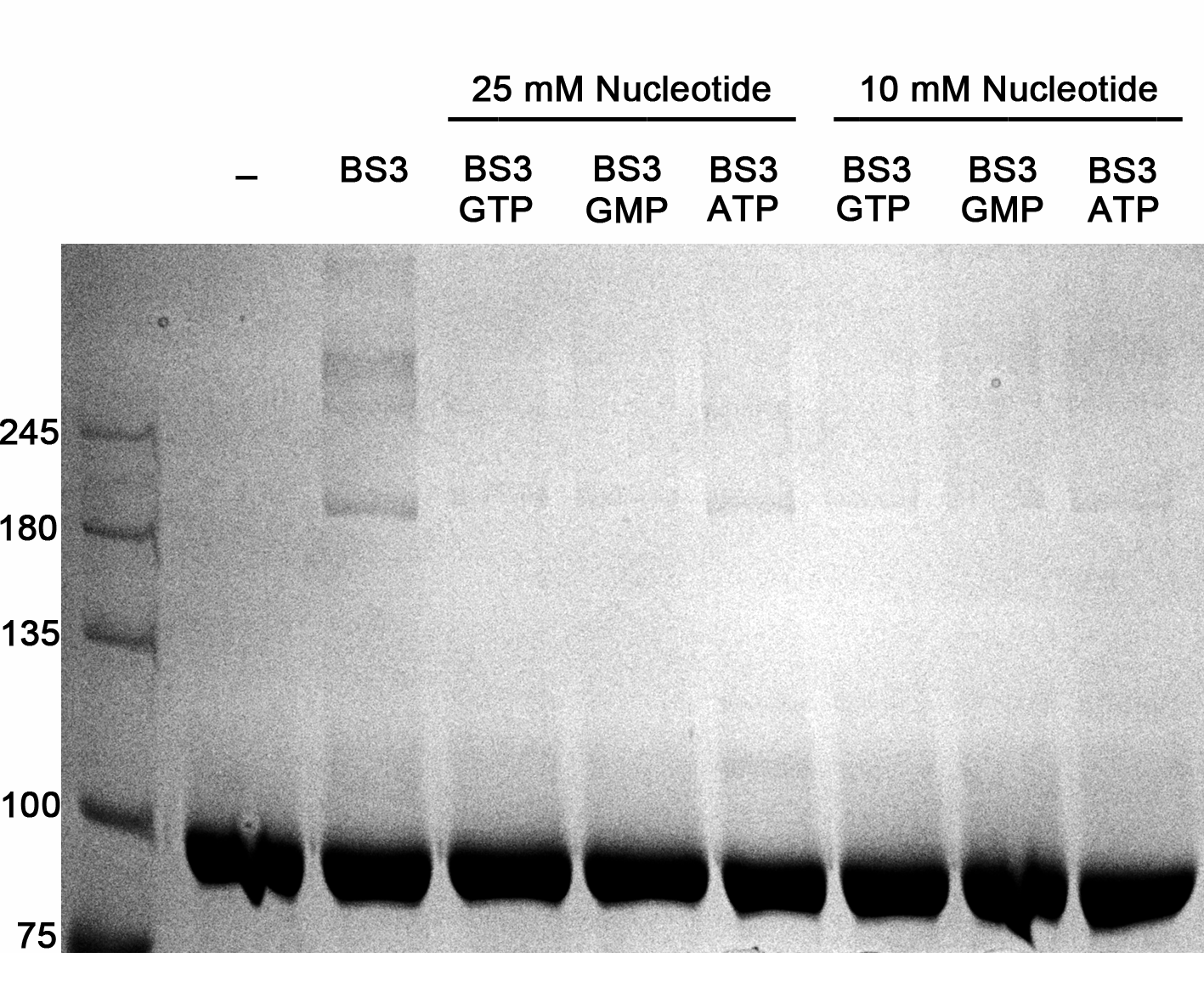

Supplement: S11 Fig — SDS-PAGE of the ZIKV NS5 wild type protein (5 μM) crosslinked with 10 μM BS3 (45 min incubation), in buffer containing 20 mM HEPES pH 7.0 and 150 mM NaCl, in absence or presence of GTP, GMP and ATP at 25 mM and 10 mM nucleotide concentration. (TIF) [file ppat.1007656.s011.tif]
