# Supplementary material for: Supramolecular arrangement of the full-length Zika virus NS5
Source: PLoS Pathog. 2019 Apr 5;15(4):e1007656. doi: 10.1371/journal.ppat.1007656 (PMC6469808; doi:10.1371/journal.ppat.1007656)
Supplement: S1 Table — (DOC) [file ppat.1007656.s012.doc]

| Parameters | 5M2X | 6I7P | 5M2Z |
| --- | --- | --- | --- |
| **Data Collection**  Resolution range (Å) | 49.52 – 4.99 (5.11-4.99)^#^ | 49.52-3.98 (4.05-3.98)^#^ | 44.33-4.8 (5.06-4.80)^#^ |
| Lowest cut-off difraction limit (Å) (direction)^¶^ |  | 7.39 (0.857b* + 0.514c*) |  |
| Best diffraction limit after cut-off (Å) (direction)^¶^ |  | 3.98 (0.07a* + 0.01b* + 0.99c*) |  |
| Space group | P2_1_2_1_2_1_ | P2_1_2_1_2_1_ | P6_5_ |
| Unit cell parameters  a, b, c (Å)  α, β, γ (º) | 191.06 192.06 407.23  90 90 90 | 191.06 192.06 407.23  90 90 90 | 234.58 234.58 406.12  90 90 120 |
| Measured reflections | 1,962,662 (224755) | 3,890,662 (143079) | 202753(27952) |
| Unique reflections | 33882(4342) | 128656 (6433) | 61422 (8906) |
| Rmerge^§^ | 0.750(1.694) | 0.963(1.941) | 0.159 (1.495) |
| Rpim^◊^ | 0.097(0.273) | 0.175 (0.402) | 0.104(1.005) |
| Multiplicity | 57.9(51.8) | 30.2(22.2) | 3.3 (3.1) |
| Completeness (%) | 99.5(97.3) | 99.6 (92.0) | 99.7 (99.4) |
| Mean I/Sigma | 5.8(3.6) | 5.2(2.0) | 5.9 (1.1) |
| **Refinement** |  |  |  |
| Resolution range | 49.52 – 4.99 | 49.52-3.98 | 44.07-4.80 |
| Rwork^†^ | 0.2270 | 0.2805 | 0.2410 |
| Rfree^‡^ | 0.2760 | 0.2805 | 0.2660 |
| Protein residues | 5297 | 5297 | 5298 |
| Ligands | 14 | 14 | 14 |
| Rms bond lenghts (Å) | 0.0112 | 0.004 | 0.0104 |
| Rms angles (º) | 1.3744 | 0.665 | 1.2571 |
| Ramachandran plot (%) |  |  |  |
| Favored | 94.66 | 94.53 | 95.67 |
| Allowed | 4.88 | 3.88 | 3.76 |
| Outliers | 0.45 | 0.11 | 0.57 |

**Table S1.** Data collection and Refinement Statistics

^#^ The number in parentheses refers to the last (highest) resolution shell.

^¶^ These statistics are for data that were truncated by STARANISO.

§ Rmerge = Σ|I j - <I>| / Σ I j where I j is the intensity of an individual reflection and <I> is the average intensity of that reflection.

† Rwork = Σhkl ||Fobs(hkl)|—|Fcalc(hkl)|| / Σhkl |Fobs(hkl)|, where Fobs and Fcalc are the structure factors, deduced from measured intensities and

calculated from the model, respectively.

‡ Rfree = as for Rwork but for 5% of the total reflections chosen at random and omitted from refinement

^◊^ Rpim = ∑ hkl √1/n−1∑∣I_i_ (hkl)−̄I (hkl)∣ / ∑ ∑ I_i_ (hkl)
