# Supplementary material for: Supramolecular arrangement of the full-length Zika virus NS5
Source: PLoS Pathog. 2019 Apr 5;15(4):e1007656. doi: 10.1371/journal.ppat.1007656 (PMC6469808; doi:10.1371/journal.ppat.1007656)
Supplement: S2 Table — (DOCX) [file ppat.1007656.s013.docx]

**Table S2. Small Angle X-ray Scattering parameters**

| **Conc.**  **(mg/ml)** | **Io/c** | **Rg (nm)** | **Dmax (nm)** | **MW^Exp^ (kDa)** |
| --- | --- | --- | --- | --- |
| 0.5 | 82 | 4.7 | 19 | 114 |
| 1 | 91 | 4.8 | 19 | 118 |
| 2 | 103 | 5.3 | 22 | 139 |
| 4 | 124 | 5.8 | 23 | 182 |
| 6 | 121 | 6.1 | 23 | 206 |
